# Comparison of the penile microbiome in infant male circumcision: Mogen clamp versus Shangring

**DOI:** 10.1016/j.ebiom.2024.105216

**Published:** 2024-06-25

**Authors:** Juan E. Salazar, Daniel E. Park, Nahid Punjani, Tony Pham, Maliha Aziz, Godfrey Kigozi, Ronald H. Gray, Stephen D. Kiboneka, Marc Goldstein, Philip S. Li, Richard Lee, Cindy M. Liu

**Affiliations:** a Antibiotic Resistance Action Center, Department of Environmental and Occupational Health, Milken Institute School of Public Health, George Washington University, Washington, DC, 20052, USA; b Department of Urology, Weill Cornell Medicine of Cornell University, New York-Presbyterian Hospital, New York, NY, USA; c Rakai Health Sciences Program, Uganda Virus Research Institute, Entebbe, Uganda; d Department of Epidemiology, Johns Hopkins University, Bloomberg School of Public Health, Baltimore, USA

**Keywords:** Infant penile microbiome, Infant coronal sulcus microbioime, Early infant male circumcision (EIMC), Anaerobe, Mogen clamp, Shangring, Uropathogen, Tetanus

## Abstract

**Background:**

This study aimed to characterise the infant penile (coronal sulcus) microbiome and the effects of early infant male circumcision (EIMC), following a standard surgical method (Mogen Clamp) and a non-surgical alternative (ShangRing).

**Methods:**

We collected coronal sulcus swabs at baseline and on days 7 and 14 post-circumcision from infants assigned to receive EIMC by Mogen Clamp (n = 15) or ShangRing (n = 15), in a randomised trial in Rakai and Kakuuto, Uganda. We used 16S rRNA gene-based sequencing and broad-coverage qPCR to characterise the infant penile microbiome and assess the effects of EIMC in both study arms.

**Findings:**

Prior to EIMC, the infant penile microbiome had a mixture of facultative and strict anaerobes. In both study arms, EIMC caused penile microbiome proportional abundance changes characterised by decreases in penile anaerobes [ShangRing *Prevotella*: −15.0%, (SD = 19.1); Mogen clamp *Prevotella*: −3.6% (11.2); ShangRing *Veillonella*: −11.3% (17.2); Mogen clamp *Veillonella*: −2.6% (11.8)] and increases in skin-associated facultative anaerobes [ShangRing *Corynebacterium*: 24.9%, (22.4); Mogen clamp *Corynebacterium*: 4.7% (21.3); ShangRing *Staphylococcus*: 21.1% (20.5); Mogen clamp *Staphylococcus*: 18.1% (20.1)]. *Clostridium tetani* was not detected during the study.

**Interpretation:**

Mogen Clamp and ShangRing EIMC both changed the composition of the infant penile microbiome by reducing the proportional abundances of anaerobes and uropathogens, which is consistent with medical male circumcision findings in adults. *C. tetani* was not increased by either EIMC method.

**Funding:**

10.13039/100000865Bill and Melinda Gates Foundation.

**Research in context**

**Evidence before this study**

Medical male circumcision in infants and adults are both associated with protection against infectious diseases. While we know that the health benefits of medical male circumcision in adults are conferred at least in part through microbiome changes, less is known about the infant penile microbiome, or the effects of early infant male circumcision (EIMC). Additionally, determining the safety of non-surgical approaches, such as the ShangRing may be important for expanding treatment access in resource-limited settings. A previously used single-use non-surgical device (PrePex), was linked to an increased risk of tetanus (*Clostridium tetani)* infection.

We searched MEDLINE without language restrictions for articles published before August 2023. Search terms included combinations of “infant”, “pediatric, “penile”, “microbiome”, “microbiota”, “microflora”, “circumcision”, and related terms. The search identified only two studies which directly assessed the infant penile microbiome before and after circumcision. One study (Günsar et al., 2004) was cultured-based and focused on bacteria collected from the periurethral and glanular sulcus regions. The study reported an increase in commensal skin bacteria and a decrease in pathogenic bacteria post-circumcision. A second study (Mishra et al., 2023) analysed 11 paired periurethral samples through 16s rRNA sequencing and ITS to identify if predicted inflammatory pathways were associated with particular bacterial or fungal profiles post-circumcision. Limitations of both studies included utilization of a single (surgical) EIMC method, small sample size, and limited post-circumcision follow-up.

**Added value of this study**

Our study contributes to our knowledge on the infant penile microbiome and evaluates the impact of a non-surgical EIMC method on the infant penile microbiome, as compared to the standard surgical method. Our study was performed to meet the need for non-surgical alternatives to medical male circumcision that can be deployed in resource-limited settings. By examining the microbiological profile post-circumcision, we assessed potential risks that may be associated with the ShangRing device. This research may have implications for clinical and public health practice, particularly in regions where voluntary medical male circumcision programs including EIMC are utilised for HIV prevention.

**Implications of all the available evidence**

The results of our study suggest that both surgical and non-surgical EIMC result in similar post-circumcision microbiome dynamics. Generally, anaerobic bacteria and uropathogenic taxa decrease following circumcision. The bacteria which causes tetanus, *Clostridium tetani*, was not detected at any point in our cohorts. A favourable safety profile of the ShangRing could encourage its adoption as a method for infant circumcision in resource-limited settings. This study advances our understanding of circumcision on the infant penile microbiome which may inform clinical practices, policy considerations, and future research directions, ultimately contributing to safer and more effective circumcision procedures.

## Introduction

Little is known regarding the genital microbiome in infants. Similarly, the effect of early infant male circumcision (EIMC) on the infant penile microbiome is also not well understood. Earlier studies suggest that the penile microbiome in uncircumcised male infants is likely comprised of uropathogens that are reduced by EIMC. Specifically, EIMC was shown to reduce the incidence of urinary tract infections, decrease fungal abundance, and decrease potential uropathogens such as *Escherichia coli* in the preputial space in infants.^1^, ^2^, ^3^ Given its various health benefits, EIMC is recommended for preventing urinary tract infections in male infants^4^^,^^5^

Early infant male circumcision remains reliant on surgical methods such as Mogen clamp, which require specialised personnel and equipment. However, there is a demand for effective non-surgical options, such as medical devices, which are low-cost and have high acceptance among parents.^6^, ^7^, ^8^, ^9^ Currently, the ShangRing, which received prequalification approval from WHO in 2015 and 2019, is the main non-surgical alternative.^10^ The ShangRing is a single-use device that may be used in infants, adolescents, and adults with topical or injectable anesthesia. It requires no suturing, which could simplify device implementation.^11^ Hemostasis is achieved using two concentric rings which compress the wound after the foreskin is removed. The device remains in place for 7 days before removal at a follow-up visit. ShangRing circumcision has been shown to be effective and safe from infancy to adulthood.^12^, ^13^, ^14^

In the current study, we characterised the penile microbiome in uncircumcised male infants. We further assessed the impact of EIMC on the infant penile coronal sulcus microbiome, comparing two different EIMC methods: Mogen Clamp and ShangRing. We hypothesised that both EIMC approaches would significantly decrease anaerobes and uropathogens in the infant penile coronal sulcus microbiome.

## Methods

### Ethics statement

Participant's parents or legal representatives provided written informed consent for infant participation in the trial. Human subject research approvals were obtained in Rakai and Kakuuto, Uganda (WCMC IRB#: IRB00009417). This was determined to be non-human subjects research by the George Washington institutional review board. All experiments and samplings were carried out in accordance with ethical and biosafety protocols.

### Study design

The full randomised controlled trial comparing ShangRing and Mogen clamp for early infant male circumcision in eastern sub-Saharan Africa was described in detail previously (protocol available).^14^ Briefly, eligible participants were healthy male infants (aged <60 days) with a gestational age of at least 37 weeks and a birthweight of at least 2.5 kg. Infants with perinatal illnesses requiring treatment, congenital genitourinary abnormalities requiring surgery, allergies to known anaesthetic components, or a family history of bleeding disorders were excluded. Enroled infants were randomly assigned (1:1) to undergo EIMC by either Mogen clamp or the ShangRing. Participants were randomised using a computer-generated allocation sequence. A convenience sample was taken from the full trial to determine the impact of male circumcision on the infant penile microbiome.

### Sample collection

We collected coronal sulcus samples from infants assigned to receive ShangRing circumcision, (n = 15) and Mogen Clamp circumcision (n = 15) in Rakai and Kakuuto, Uganda. To assess infant penile microbiome changes at timepoints relevant to post-circumcision wound healing and potential wound infection, we collected samples immediately prior to EIMC, 7-, and 14-days post-EIMC, prior to expected complete wound healing. Samples were collected using saline moistened swabs which were rolled over the coronal sulcus. Generally, baseline and follow-up specimens were collected in the mornings between 6 am and 12 pm. Swabs were placed in a custom transport media (1 mL) comprised of sterile PBS with 1% protease-free BSA (A3059, Sigma–Aldrich) and 1X protease inhibitor (11836170001, Sigma–Aldrich), then stored at −80 °C until analysis.

### Infant penile microbiome characterization

First, we extracted total DNA from 80 μL of undiluted swab eluent using a combination of enzymatic and chemical lysis. Briefly, each sample was treated with an enzymatic cocktail containing 122 μL Tris–EDTA, 50 μL 10 mg/mL lysozyme (L6876-1G, Sigma–Aldrich), 4 μL 25 KU/mL mutanolysin (M4782-5KU, Sigma–Aldrich), and 3 μL 4 U/μL lysostaphin (SAE0091-2 MG, Sigma–Aldrich) at 37 °C for 1 h. DNA was isolated using a MagMax DNA Multi-Sample Ultra 2.0 Kit with 80 μL final elution volume, following manufacturer instructions.

Next, we characterised the penile microbiome by 16S rRNA gene-based broad-range real-time PCR and sequencing. A modified protocol from Fadrosh et al., 2014 with forward (341F) and reverse (786R) primers from Liu et al., 2012 was used to amplify the V3–V4 region of the 16S rRNA gene.^15^^,^^16^ Sequencing was performed on MiSeq platform using MiSeq Reagent Kit v3 (600-cycle).

During data processing, primer sequences were removed from the amplicon sequences using cutadapt v2.4 and the resultant sequences were quality-trimmed using Trimmomatic v0.39.^17^^,^^18^ DADA2 v1.10 modules were used for reads-filtering, chimera check, and inferred error models to identify Amplicon Sequence Variants (ASVs).^19^ The ASVs were classified at each taxonomic level at 80% bootstrap confidence level using the Naïve Bayesian Classifier (v.2.12).^20^ Classification results for each sample were enumerated to generate an abundance matrix for analysis. Sequence data for this study can be accessed at SRA project number PRJNA783013. Additional details can be found at https://github.com/araclab/mb_analysis.

### Statistical analysis

Coronal sulcus microbiota were summarised based on prevalence, proportional abundance, and absolute abundance. Prevalence was calculated as the number of participants in a study arm who were positive for a given taxon, divided by the total number of participants in the study arm. The proportional abundance of each taxon was measured as the number of 16S rRNA gene sequences assigned to a taxon in a sample, divided by the total number of sequences for that sample—proportional abundance is presented as a percentage. Using our qPCR and sequencing outputs, absolute abundance was also calculated as the product of proportional abundance and total penile bacterial load (measured by qPCR as total 16S rRNA gene copies per coronal sulcus swab). We presented mean proportional abundance (with standard deviation) as a percentage and median absolute abundances (with interquartile range) for each timepoint and treatment arm. All statistical analyses utilised non-parametric significance tests. Additional summaries of median proportional abundances as percentages with interquartile range can be found in Tables S5 and S6. To control the false discovery rate (FDR), the Benjamini Hochberg procedure was applied to adjust *P*-values.

Overall microbiota composition was visualised using nonmetric multidimensional scaling (nMDS) and compared between groups using permutational multivariate analysis of variance (PerMANOVA) with hellinger-transformed proportional abundance data. Bacterial prevalences were compared using Chi–Square tests. Changes in prevalence were calculated and within arm prevalence changes were tested for significance using McNemar's test. For between group comparisons, both proportional abundance and absolute abundance significance testing were done using omnibus Kruskal–Wallis tests followed by Mann–Whitney U tests. For within group comparisons, proportional abundance changes and absolute abundance changes over time were assessed for statistical significance using omnibus Friedman tests followed by pairwise Wilcoxon signed rank tests. Significance tests used α = 0.05. Statistical analyses were performed using GraphPad Prism Software 9.0 and R (version 4.1.1).

### Role of the funding source

The funder of the study had no role in study design, data collection, data analysis, data interpretation, or writing of the report.

## Results

### Participant enrolment and demographics

**Fig. 1:**
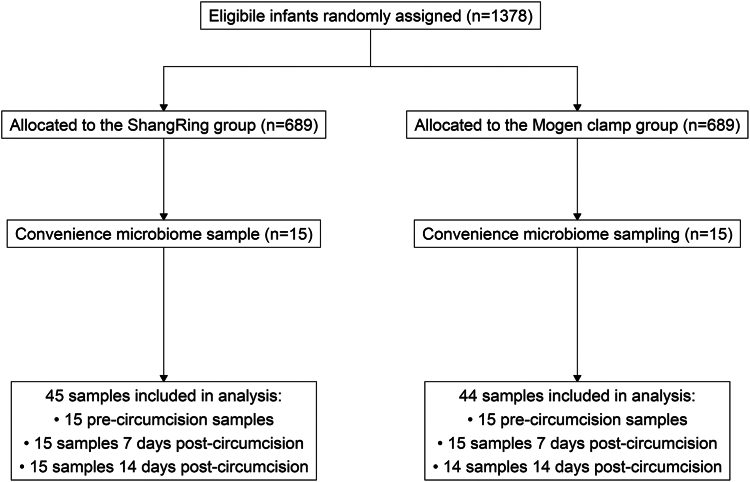
**Participant enrolment into the microbiome substudy**. A convenience sample was taken from a broader non-inferiority randomised controlled trial conducted from September 17, 2018 to December 20, 2019. Participants for the microbiome substudy were recruited from April 24, 2019 to June 13, 2019 and samples were collected from April 24, 2019 until June 27, 2019.

Between April 24, 2019, and June 13, 2019, a total of 30 infants were enroled as a convenience sample of a non-inferiority randomised controlled trial.^14^ No participants in our study were excluded or lost to follow-up, however one specimen collected from a participant in the Mogen Clamp group was excluded from analyses due to insufficient sample (Fig. 1). Participants were evenly split between clinical sites in Rakai and Kakuuto (n = 15 in each site). Participants in both study arms were similar in age (ShangRing median and interquartile range [IQR]: 27 days [16.5–41]; Mogen Clamp median and IQR: 19 days [11.0–41.5]), weight, and maternal characteristics, including age, ethnic group, and delivery method (Table 1).

**Table 1:** Characteristics of infants and parents or legally authorised representatives by treatment arm at enrolment.

	Median (IQR); n (%)	
	ShangRing (n = 15)	Mogen clamp (n = 15)
**Participant age (Days)**	27.0 (16.5, 41.0)	19.0 (11.0, 41.5)
**Weight (kg)**	3.8 (3.4, 4.4)	4.1 (3.4, 4.6)
**Age of parent or legal representative (Years)**	24.0 (21.5, 26.5)	25.0 (22.5, 27.5)
**Clinical site**		
Kakuuto	7.0 (46.7)	8.0 (53.3)
Rakai	8.0 (53.3)	7.0 (46.7)
**Ethnic group**		
Baganda	10.0 (66.7)	10.0 (66.7)
Banyankole	4.0 (26.6)	4.0 (26.6)
Other	1.0 (6.7)	1.0 (6.7)
**Method of infant delivery**		
Spontaneous vaginal delivery	13.0 (86.7)	11.0 (73.3)
Caesarean section	2.0 (13.3)	4.0 (26.7)

### Penile coronal sulcus microbiome in uncircumcised infants

At baseline, participants had similar penile microbiome profiles with a high prevalence of facultative anaerobic bacteria associated with skin, such as *Corynebacterium* and *Staphylococcus*, and with urinary tract pathogens, such as *Escherichia*. Participants also had a high prevalence of anaerobes associated with the skin, gut, and genital tract, including *Prevotella, Anaerococcus*, *Peptoniphilus*, *Finegoldia*, and *Bifidobacterium* (Table 2; Table S1). Overall, the penile microbiome of uncircumcised infants comprised uropathogens and bacteria from multiple body sites, including the skin, gut, and genital tract.

**Table 2:** Prevalence and proportional abundance of the 20 most prevalent penile bacteria in uncircumcised infants by treatment arm at enrolment.

Genus	Oxygen tolerance and pathogenicity^a^	Body sites^b^	Prevalence in group, n (%)		Average proportional abundance in group, % (SD)	
			ShangRing (n = 15)	Mogen clamp (n = 15)	ShangRing (n = 15)	Mogen clamp (n = 15)
Corynebacterium	FAN	Skin, genital	15 (100)	15 (100)	15.7 (16.1)	14.9 (15.0)
Staphylococcus	FAN	Skin, genital	13 (86.7)	12 (80.0)	7.3 (7.1)	13.8 (19.0)
Prevotella	AN	Genital, gut	13 (86.7)	8 (53.3)	16.4 (19.2)	5.3 (9.8)
Veillonella	AN	Genital, gut	10 (66.7)	7 (46.7)	11.4 (17.3)	4.1 (9.6)
Finegoldia	AN	Skin, genital, gut	11 (73.3)	11 (73.3)	1.3 (1.3)	3 (6.4)
Porphyromonas	AN	Genital	8 (53.3)	2 (13.3)	0.9 (1.3)	2.8 (8.4)
Peptoniphilus	AN	Genital, gut	10 (66.7)	8 (53.3)	1.6 (2.2)	0.9 (1.4)
Anaerococcus	AN	Skin, genital, gut	12 (80.0)	12 (80.0)	0.8 (1.0)	2.0 (2.9)
Peptostreptococcus	AN	Genital	11 (73.3)	7 (46.7)	1.6 (2.8)	0.8 (1.1)
Dialister	AN	Genital	5 (33.3)	5 (33.3)	0.4 (0.7)	0.3 (0.7)
Anaeroglobus	AN	Genital	1 (6.7)	2 (13.3)	0.04 (0.2)	3.2 (11.7)
Escherichia	FAN/UP	Gut	9 (60.0)	10 (66.7)	6.4 (15.6)	2.2 (4.9)
Enterococcus	FAN/UP	Gut	9 (60.0)	6 (40.0)	1.6 (3.8)	0.2 (0.3)
Klebsiella	FAN/UP	Gut	5 (33.3)	5 (33.3)	0.3 (0.6)	6.2 (15.0)
Pseudomonas	FAN/AN/MAE/UP	Skin, gut	11 (73.3)	12 (80.0)	2.2 (3.8)	1.5 (2.4)
Bifidobacterium	AN	Genital, gut	14 (93.3)	10 (66.7)	2.5 (4.0)	4.1 (9.1)
Streptococcus	FAN	Gut	12 (80.0)	9 (60.0)	7.5 (11.7)	2.4 (3.5)
Lactobacillus	FAN/AN/MAE	Skin, genital, gut	8 (53.3)	6 (40.0)	0.4 (0.6)	5.1 (18.8)
Actinomyces	FAN	Gut	9 (60.0)	7 (46.7)	1.5 (2.8)	0.9 (1.6)
Clostridium	AN	Gut	4 (26.7)	2 (13.3)	0.04 (0.1)	0.01 (0.04)

a AN, obligate anaerobe; FAN, facultative anaerobic; MAE, microaerophilic; UP, uropathogen.

b Taxa are commonly identified on these human body sites.

*Corynebacterium* and *Staphylococcus* were the most proportionally abundant taxa of the uncircumcised infant penile microbiome. On average, *Corynebacterium* comprised 15.7% (SD = 16.1%) and 14.9% (15.0%) of the uncircumcised infant penile microbiome in the ShangRing and Mogen Clamp arms, respectively. *Staphylococcus* was also abundant (ShangRing: 7.3% (7.1%), Mogen clamp: 13.8% (19.0%)). *Prevotella, Veillonella*, *Streptococcus*, *Escherichia*, *Bifidobacterium*, and *Lactobacillus* spp. were also detected at an average proportional abundance of at least 5%. The remaining taxa present on the coronal sulcus were detected with an average proportional abundance below 2.5% (Table 2; Table S1). The overall penile microbiome composition was not significantly different between the two study arms at baseline (PerMANOVA F statistic = 1.3; *P* = 0.22).

### ShangRing and Mogen clamp both reduce penile anaerobes and uropathogens

Post-EIMC, penile anaerobes decreased in both study arms. However, infants circumcised using ShangRing had broader and more persistent decreases in anaerobes and uropathogens than those circumcised using Mogen Clamp. Participants in both arms showed reductions in the prevalence of anaerobes and uropathogens 7 days post-EIMC. *Corynebacterium* remained ubiquitous post-EIMC (Table 3).

**Table 3:** Change in prevalence and proportional abundance of the 20 most prevalent penile bacteria after circumcision by treatment arm at day 7 and 14 of the trial.

Genus	Change in prevalence, %^a^						Change in proportional abundance in group, % (SD)^b^^,^^c^					
	Day 0–Day 7		Day 7–Day 14		Day 0–Day 14		Day 0–Day 7		Day 7–Day 14		Day 0–Day 14	
	ShangRing (n = 15)	Mogen clamp (n = 15)	ShangRing (n = 15)	Mogen clamp (n = 14)	ShangRing (n = 15)	Mogen clamp (n = 14)	ShangRing (n = 15)	Mogen clamp (n = 15)	ShangRing (n = 15)	Mogen clamp (n = 14)	ShangRing (n = 15)	Mogen clamp (n = 14)
Corynebacterium	0.0	0.0	0.0	0.0	0.0	0.0	4.0 (23.3)	−1.3 (15.7)	20.9 (24.7)	7.2 (22.6)	24.9 (22.4)∗	4.7 (21.3)
Staphylococcus	13.3	20.0	0.0	0.0	13.3	20.0	35.5 (29.4)∗∗	42.5 (37.8)∗	−14.3 (34.1)	−23.8 (38.6)	21.1 (20.5)∗∗	18.1 (20.1)
Prevotella	−20.0	−13.3	−20.0	31.4	−40.0	18.1	−12.1 (16.4)	−3.7 (10.9)	−2.9 (11.2)	0.3 (3.5)	−15.0 (19.1)∗	−3.6 (11.2)
Veillonella	−33.4	−33.4	−6.6	36.7	−40.0	3.3	−10.1 (18)	−4.1 (9.6)	−1.2 (4.4)	1.8 (4.8)	−11.3 (17.2)∗	−2.6 (11.8)
Finegoldia	26.7	13.4	0.0	13.3	26.7	26.7	1.3 (2.6)	−0.6 (6.8)	0.5 (5.1)	3.8 (7.3)	1.7 (4.8)	3.1 (10.5)
Porphyromonas	0.0	20.0	−20.0	16.7	−20.0	36.7	−0.1 (2.2)	−1.6 (7.7)	1.6 (4.8)	3.3 (8.5)	1.5 (5.5)	1.6 (6.2)
Peptoniphilus	33.3	20.0	−6.7	12.4	26.6	32.4	0.3 (2.9)	0.2 (2.5)	−0.3 (3.1)	1.5 (2.5)	0.0 (3.2)	1.7 (3.6)
Anaerococcus	20.0	0.0	−6.7	12.9	13.3	12.9	4.0 (5.8)	0.1 (3.6)	−2.7 (5.1)	2.7 (4.5)	1.4 (3)	2.9 (5.7)
Peptostreptococcus	6.7	0.0	−40.0	10.4	−33.3	10.4	1.7 (7.3)	1.1 (3.6)	−2.4 (5.8)	−0.2 (4.8)	−0.7 (3.5)	0.9 (2.3)
Dialister	−20.0	−20.0	13.4	8.1	−6.7	−11.9	−0.4 (0.7)	−0.3 (0.8)	0.1 (0.3)	0.0 (0.1)	−0.3 (0.8)	−0.2 (0.8)
Anaeroglobus	−6.7	−13.3	0.0	0.0	−6.7	−13.3	0.0 (0.1)	−3.2 (11.7)	0.0 (0.0)	0.0 (0.0)	0.0 (0.1)	−3.4 (12.1)
Escherichia	−40.0	−20.0	0.0	3.3	−40.0	−16.7	−6.3 (15.6)	−0.9 (5.3)	0.9 (3.5)	−0.3 (4.1)	−5.4 (16.4)	−1.2 (5.6)
Enterococcus	−46.7	−26.7	6.7	43.8	−40.0	17.1	−1.6 (3.8)	−0.1 (0.5)	0.1 (0.3)	0.3 (0.8)	−1.5 (3.9)∗	0.2 (0.8)
Klebsiella	−20.0	6.7	0.0	10.0	−20.0	16.7	−0.2 (0.6)	−1.9 (19.7)	0.1 (0.3)	−4.1 (11.9)	−0.2 (0.6)	−6.1 (15.5)
Pseudomonas	−40.0	−53.3	−13.3	23.3	−53.3	−30.0	−2.1 (3.9)	−1.3 (2.5)	0.0 (0.5)	1.4 (5.4)	−2.1 (3.8)∗	0.0 (6.4)
Bifidobacterium	−26.6	0.0	6.6	26.2	−20.0	26.2	5.0 (11.0)	−3.3 (9.4)	−3.1 (14.9)	11.7 (22.1)	1.9 (10.6)	8.0 (24.8)
Streptococcus	−6.7	13.3	−20.0	−1.9	−26.7	11.4	−5.3 (13.6)	0.1 (6.2)	−0.1 (7.4)	−0.9 (3.5)	−5.4 (13.8)	−0.9 (5.1)
Lactobacillus	−20.0	−33.3	−20.0	7.6	−40.0	−25.7	−0.3 (0.6)	−5 (18.8)	0.0 (0.1)	0.0 (0.2)	−0.3 (0.6)	−0.2 (0.7)
Actinomyces	−6.7	−20.0	−33.3	9.0	−40.0	−11.0	−1.3 (2.7)	−0.5 (1.4)	−0.1 (0.3)	−0.2 (0.8)	−1.4 (2.7)	−0.8 (1.7)
Clostridium	−20.0	0.0	0.0	−13.3	−20.0	−13.3	0.0 (0.1)	0.0 (0.1)	0.2 (0.7)	0.0 (0.0)	0.2 (0.8)	0.0 (0.0)

∗*P* < 0.05. ∗∗*P* < 0.01.

a Change in prevalence is a percent change calculated by the change in prevalence observed in a single treatment group over time. Significance testing for within arm changes was done using McNemar's test and adjusted for multiple comparisons using Benjamini-Hochberg correction.

b Change in Proportional abundance is a percent change calculated by the change in proportional abundance observed in a single treatment group over time. Significance testing for within arm changes was done using Wilcoxon matched pairs signed-rank test and adjusted for multiple comparisons using Benjamini-Hochberg correction.

c Significance testing for between arm changes was done using Mann–Whitney U test and adjusted for multiple comparisons using Benjamini-Hochberg correction.

While anaerobes and uropathogens continued to decrease in prevalence from day 7 to day 14 post-EIMC among ShangRing arm participants, some increased in prevalence in the Mogen Clamp arm. Specifically, 14 days post-EIMC, the prevalence of *Prevotella* (change in prevalence: −40.0%)*, Veillonella* (−40.0%)*, Escherichia* (−40.0%)*, Enterococcus* (−40.0%)*, Pseudomonas* (−53.3%), *Lactobacillus* (−40.0%), and *Actinomyces* (−40.0%) spp. had all decreased in the ShangRing arm (n = 15); however, *Finegoldia* (26.7%), *Porphyromonas* (36.7), *Peptoniphilus* (32.4) spp. increased in prevalence among Mogen Clamp arm participants (n = 14) (Table 3; Table S2). Similar trends were observed in the proportional abundances of anaerobes and uropathogens. At 14 days post-EIMC, ShangRing arm participants had significantly decreased proportional abundances of *Prevotella* (mean change in proportional abundance: −15% (SD = 19.1%)), *Veillonella* (−11.3% (17.2%)), *Enterococcus* (−1.5% (3.9%)), and *Pseudomonas* (−2.1% (3.8%)) spp. (Table 3). Mogen Clamp arm participants also had decreases in these specific taxa, but these changes were not statistically significant.

**Fig. 2:**
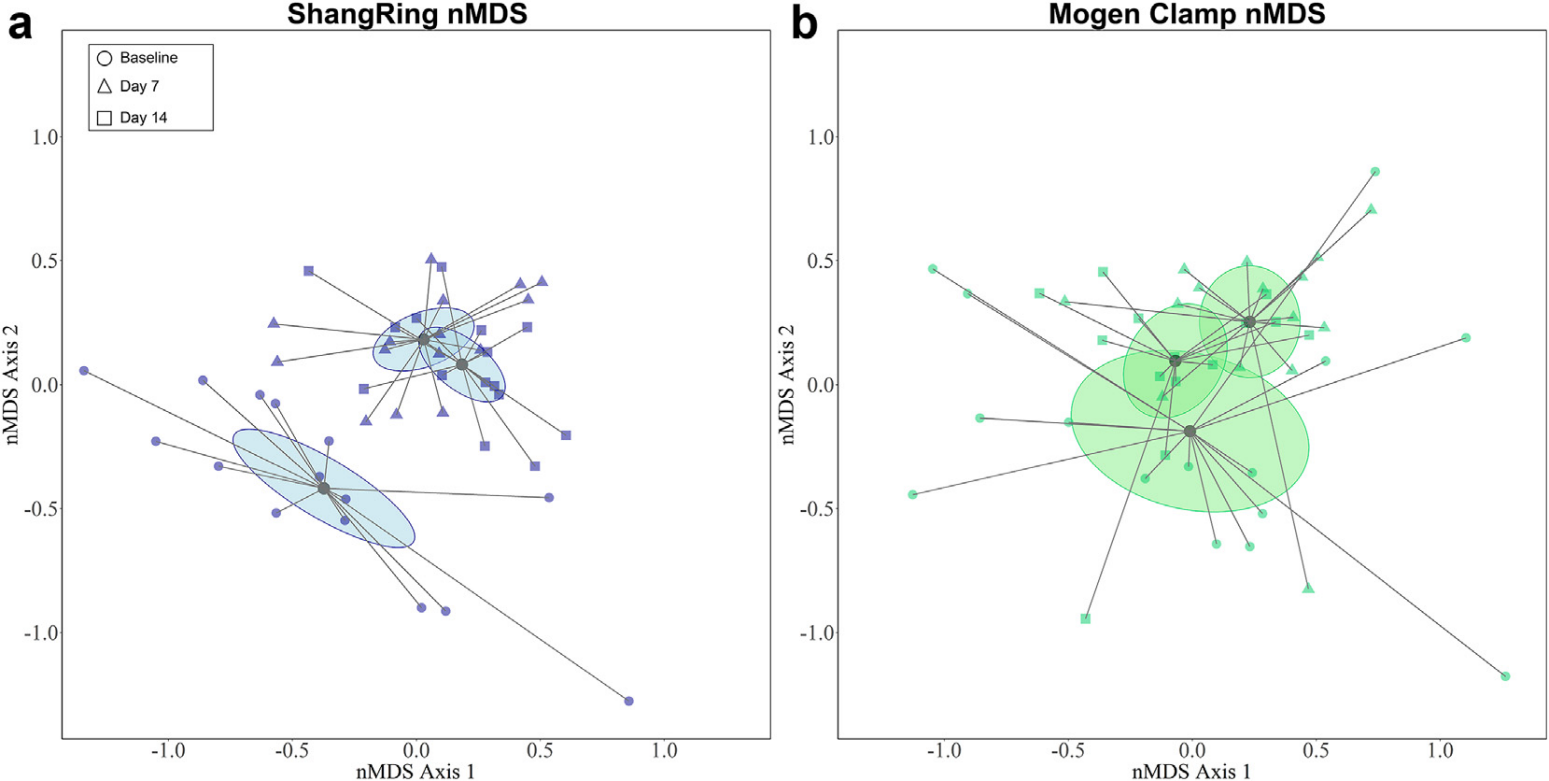
**Nonmetric multidimensional scaling (nMDS) ordination plot of participant coronal sulcus microbiome in ShangRing (a) and Mogen Clamp (b) participants.** Each point represents an individual's microbiome at a given time point and the distance between points denotes similarities between samples. Circular symbols represent baseline samples (ShangRing n = 15, Mogen clamp n = 15), triangluar symbols represent samples taken at day 7 (ShangRing n = 15, Mogen clamp n = 15), and square symbols represent samples taken at day 14 (ShangRing n = 15, Mogen clamp n = 14). Centroids and 95% confidence ellipses for each treatment arm and time point are included. Overall microbiota composition was visualised using nMDS and groups were compared using permutational multivariate analysis of variance (PerMANOVA) with hellinger-transformed proportional abundance data. Within arm changes for ShangRing (PerMANOVA F statistic = 5.7, *P* < 0.001) and Mogen Clamp (PerMANOVA F statistic = 3.4, *P* < 0.001) were both significant.

There were significant changes in overall microbiome composition post-EIMC in both arms (ShangRing PerMANOVA F statistic = 5.7, *P* < 0.001; Mogen Clamp PerMANOVA F statistic = 3.4, *P* < 0.001). These microbial shifts appeared larger in the ShangRing arm (Fig. 2a) than in the Mogen Clamp arm (Fig. 2b). *Corynebacterium* and *Staphylococcus* were the predominant coronal sulcus bacteria in both study arms post-EIMC. This was driven by immediate increases in *Staphylococcus* post-EIMC by day 7 [ShangRing: mean change in proportional Abundance = 35.5% (SD = 29.4%), *P* = 0.006; Mogen Clamp: 42.5% (37.8), *P* = 0.01] (Table 3; Table S3). However, between day 7 and day 14 post-MC, *Staphylococcus* decreased while *Corynebacterium* increased in proportional abundance in both treatment arms, suggesting a post-EIMC bacterial succession pattern (Table 3, Table S3). Overall, the changes in mean proportional abundance between both arms were similar in direction and magnitude. ShangRing participants had greater increases in *Corynebacterium* [24.7% (SD = 22.4%); Mogen Clamp: 4.7% (21.3%)] and greater decreases in *Prevotella* [−15.0% (19.1%); Mogen Clamp: −3.6% (11.2%)] and *Veillonella* [−11.3% (17.2%); Mogen Clamp: −2.6% (11.8%)] compared with Mogen clamp participants after 14 days, but these differences were not significant.

### ShangRing and Mogen clamp both increase penile bacterial density and significantly increase abundance of skin-associated bacteria

**Fig. 3:**
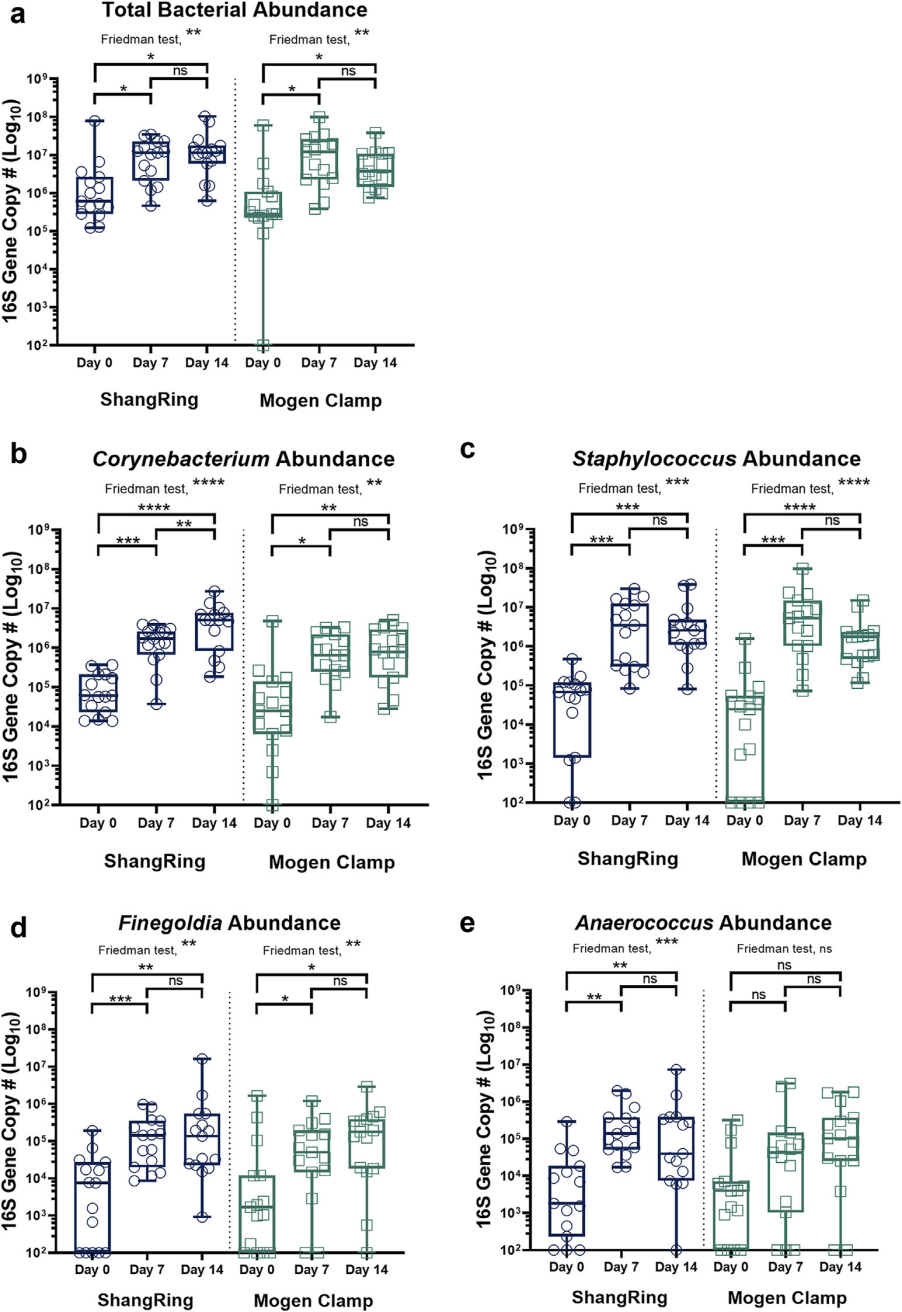
**Total bacterial and taxon-specific absolute abundance changes in the coronal sulcus by treatment arm (ShangRing = blue/left; Mogen Clamp = green/right) over time.** Box plot denotes median and interquartile range. There were statistically significant increases in total bacterial density (a), skin-associated facultative anaerobes (*Corynebacterium* (b) and *Staphylococcus* (c)), and gram-positive anaerobic cocci (*Finegoldia* (d), *Anaerococcus* (e)) in both treatment arms after 14 days. Absolute abundance changes over time within group were assessed using omnibus Friedman tests followed by pairwise Wilcoxon matched pair signed rank tests. *P*-values were corrected for the false discovery rate using the Benjamini Hochberg procedure. ∗*P* < 0.05. ∗∗*P* < 0.01. ∗∗∗*P* < 0.001. ∗∗∗∗*P* < 0.0001.

**Figure S1:**
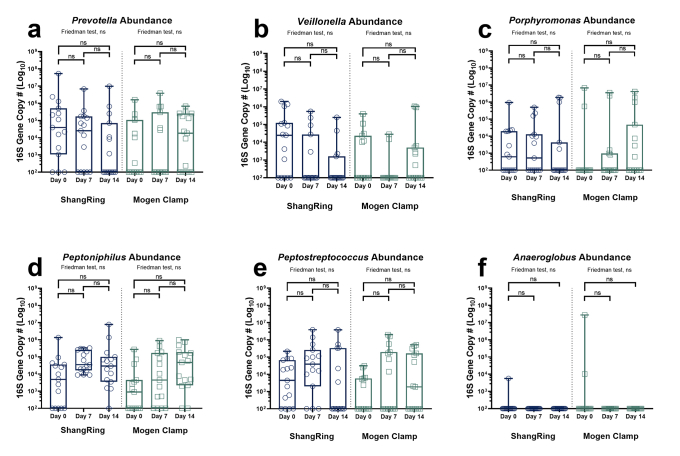
**Genital-associated anaerobe taxon-specific density changes in the coronal sulcus by treatment arm (ShangRing = blue/left; Mogen Clamp = green/right) over time.** Box plot denotes median and interquartile range with whisker signifying maximum and minimum values. Absolute abundance changes over time within-subjects were assessed using omnibus Friedman tests followed by pairwise Wilcoxon matched pair signed rank tests. P-values were corrected for the false discovery rate using the Benjamini Hochberg procedure. No significant bacterial load changes were observed in *Prevotella* (a), *Veillonella* (b), *Porphyromonas* (c), *Peptoniphilus* (d), *Peptostreptococcus* (e), or *Anaeroglobus* (f).

In both study arms, total bacterial density increased significantly post-EIMC. The baseline penile bacterial densities were similar between study arms (Mogen Clamp median: 2.8 × 10^5^ 16S rRNA gene copies per swab [IQR = 2.4 × 10^5^–9.6 × 10^5^]; ShangRing median: 6.1 × 10^5^ copies per swab [3.6 × 10^5^–2.3 × 10^6^]; *P* = 0.164) (Fig. 3a; Table S4). Seven days post-EIMC, the total penile bacterial density increased significantly and similarly in both study arms (Mogen Clamp median: 1.2 × 10^7^ copies per swab [2.4 × 10^6^–2.3 × 10^7^], *P* = 0.010; ShangRing median: 1.1 × 10^7^ copies per swab [3.0 × 10^6^–2.0 × 10^7^], *P* = 0.013; *P* = 0.838), a pattern that persisted into day 14 post-EIMC (Fig. 3a; Table S4). The significant increase in total bacterial density correlated with increased absolute abundance of *Corynebacterium*, *Staphylococcus*, *Finegoldia*, and *Anaerococcus* post-MC in both arms (Fig. 3b–e; Table S4) and limited decreases in the absolute abundances of anaerobes and uropathogens despite significant decreases in their prevalence and proportional abundance (Figures S1 and S2).

### Clostridium tetani was not detected before or after circumcision

*Clostridium* was detected in 26.7% and 13.3% of ShangRing arm and Mogen Clamp arm participants pre-EIMC, respectively (Table 2; Table S1). *Clostridium neonatale* was the only *Clostridium* species we detected in the infant penile microbiome. Among *Clostridium* carriers, *Clostridium* made up less than 1% median proportional abundance. The prevalence and abundance of *Clostridium* decreased post-EIMC in both study arms and *Clostridium tetani* was never detected in our study.

## Discussion

In a study of early infant male circumcision, we found changes to the early infant penile microbiome post-EIMC with both the ShangRing device and with standard surgical circumcision using the Mogen Clamp. Participants in both arms had comparable bacterial profiles at baseline and showed similar microbiota shifts post-circumcision. The overall bacterial abundance of the coronal sulcus significantly increased post-EIMC which was likely driven by an expansion of skin-associated facultative anaerobes, *Corynebacterium* and *Staphylococcus*. This contrasts with previous studies in adults in which the total bacterial load significantly declined following MC with longer post-MC follow up.^21^^,^^22^ The increase of *Corynebacterium* and *Staphylococcus* post-EIMC does not imply an increased risk of pathogenesis as they are common human epithelial commensals.

Although the trends observed in both circumcision groups generally mirrored each other, unexpected differences were also detected. Participants in the ShangRing arm had a broad and persistent reduction in the prevalence and proportional abundance of genital anaerobes and uropathogens compared to those in the Mogen Clamp study arm. This may indicate that ShangRing circumcision results in more effective clearance of strict anaerobic taxa, or it may be linked to post-circumcision recovery. Whereas Mogen Clamp EIMC requires no postoperative follow-up, the ShangRing device stays attached for up to 7 days before removal or spontaneous detachment. The continued presence of the ShangRing device may play a role in preventing anaerobic bacterial colonization. However, given that the number of participants was small, this study is underpowered to detect statistically significant changes in groups across all taxa of interest over time. Further, as a small convenience sample nested within a larger trial, these findings may not be generalizable and should be interpreted with caution.

Our study has several other limitations. First, the study did not include an uncircumcised control arm, so we cannot assess if the observed changes are directly attributable to EIMC itself or due to natural penile microbiome dynamics in infants. Secondly, while the observed changes in the early infant penile microbiota may be due to EIMC alteration of the penile microenvironment, they may also be due to parental behaviour or wound care after circumcision. Additionally, though this study provides important insights into the immediate microbial dynamics after EIMC, the changes observed within the first fourteen days may not reflect persistent bacterial shifts. Future studies with longer follow-up windows are required to understand the long-term dynamics post-EIMC. While infants with perinatal illnesses, congenital genitourinary abnormalities, and bleeding disorders were excluded, other conditions which may affect the host microbiome, such as atopic dermatitis or other transitory dermatoses were not reported or used as exclusion criteria. Lastly, the generalizability of our study may be limited, as our study population is composed of two nearby towns in Uganda. It is important to note, however, that this is a distinct population compared to previous reports. As such, it contributes to our understanding of diverse microbial compositions across populations.

Both circumcision methods used in this study have been shown to be safe and efficacious. Notably, however, another single-use MC device, the PrePex device, was linked to an increased risk of tetanus (*Clostridium tetani)* infection.^23^^,^^24^ While no *Clostridium tetani* sequences were detected in either treatment group post-EIMC, *Clostridium neonatale* was detected across both. Though larger studies will be needed to fully evaluate tetanus risk, our data suggest that EIMC performed with either ShangRing or Mogen Clamp do not increase the risk of tetanus infection.

To date, most penile microbiome studies have been in adults. They have consistently shown that common uropathogens, such as those from *Enterobacteriaceae*, are minor components of the penile microbiome. In uncircumcised men, the penile microbiome was found to be dominated by both gram-positive anaerobic bacteria such as *Peptoniphilus* and gram-negative anaerobic bacteria such as *Prevotella*. Like findings in this study, medical male circumcision in adults was shown to significantly reduce penile anaerobic bacteria and increase penile facultative anaerobic bacteria.^21^^,^^22^ While the direct health impacts of these microbiome changes are still to be fully elucidated, adult medical male circumcision has been demonstrated to be protective against HIV, HSV-2, and HPV in heterosexual men and against trichomoniasis and bacterial vaginosis in their female sexual partners.^25^, ^26^, ^27^, ^28^, ^29^, ^30^ Understanding post-circumcision penile microbial dynamics from infancy through to adulthood, and the various stages in between, will be key in understanding the above health impacts.

Our findings identified an increase in bacterial density after EIMC, primarily driven by increases in skin-associated facultative anaerobes, *Corynebacterium* and *Staphylococcus*. In contrast, uropathogens and penile anaerobes generally decreased after EIMC. Interestingly, these observed effect sizes were larger and more persistent in the ShangRing study arm. The reduction in uropathogens is consistent with the observed decreased risk of urinary tract infections after EIMC.^31^ *Clostridium tetani* was not detected after EIMC with Mogen clamp or using the ShangRing device. Future studies on the effects of EIMC on the penile epithelium and immune milieu, as well as the long-term effects of EIMC on the adult penile microbiome are needed to fully understand the potential health impacts of male circumcision.

## Contributors

RL, PSL, RHG, NP, MG, and CML designed the study and contributed to data analysis and manuscript preparations. SDK and GK conducted sample and data collection. JES and TP performed laboratory analysis. JES performed data analysis and drafted the manuscript. DEP and MA contributed to data analysis and bioinformatics. JES and TP verified underlying data. All authors reviewed and approved the final manuscript.

## Data sharing statement

The datasets generated and analysed for this study can be accessed at SRA project number PRJNA783013. Additional details can be found at https://github.com/araclab/mb_analysis.

## Declaration of interests

The authors declare no conflicts of interest.
