# Supplementary material for: Comparison of the penile microbiome in infant male circumcision: Mogen clamp versus Shangring
Source: eBioMedicine. 2024 Jun 25;105:105216. doi: 10.1016/j.ebiom.2024.105216 (PMC11259695; doi:10.1016/j.ebiom.2024.105216)
Supplement: Supplemental Figures and Tables [file mmc1.docx]

**Supplemental Figures and Tables – Table of Contents**

[**Figure S1**. Genital-associated anaerobe taxon-specific density changes in the coronal sulcus by treatment arm over time. 2](#_Toc158046656)

[**Figure S2.** Enteric uropathogen taxon-specific density changes in the coronal sulcus by treatment arm over time. 4](#_Toc158046657)

[**Figure S3.** Taxon-specific density changes in the coronal sulcus by treatment arm over time. 6](#_Toc158046658)

[**Table S1**. Prevalence and Proportional Abundance of the 20 Most Prevalent Penile Bacteria in Uncircumcised Infants by Treatment Arm at Enrolment 8](#_Toc160527304)

[**Table S2.** Prevalence and Change in Prevalence of the 20 Most Prevalent Penile Bacteria by Treatment Arm 7- and 14-days Post-Enrolment 10](#_Toc160527305)

[**Table S3.** Average Proportional Abundance and Change in Proportional Abundance of the 20 Most Prevalent Penile Bacteria by Treatment Arm 7- and 14-days Post-Enrolment 12](#_Toc160527306)

[**Table S4.** Median Absolute Abundance of the 20 Most Prevalent Penile Bacteria by Treatment Arm 7- and 14-days Post-Enrolment 14](#_Toc160527307)

[**Table S5.** Median Proportional Abundance of the 20 Most Prevalent Penile Bacteria by Treatment Arm 7- and 14-days Post-Enrolment 16](#_Toc160527308)

[**Table S6.** Median Change in Proportional Abundance of the 20 Most Prevalent Penile Bacteria by Treatment Arm 7- and 14-days Post-Enrolment 18](#_Toc160527309)

**Figure S1**. Genital-associated anaerobe taxon-specific density changes in the coronal sulcus by treatment arm over time.


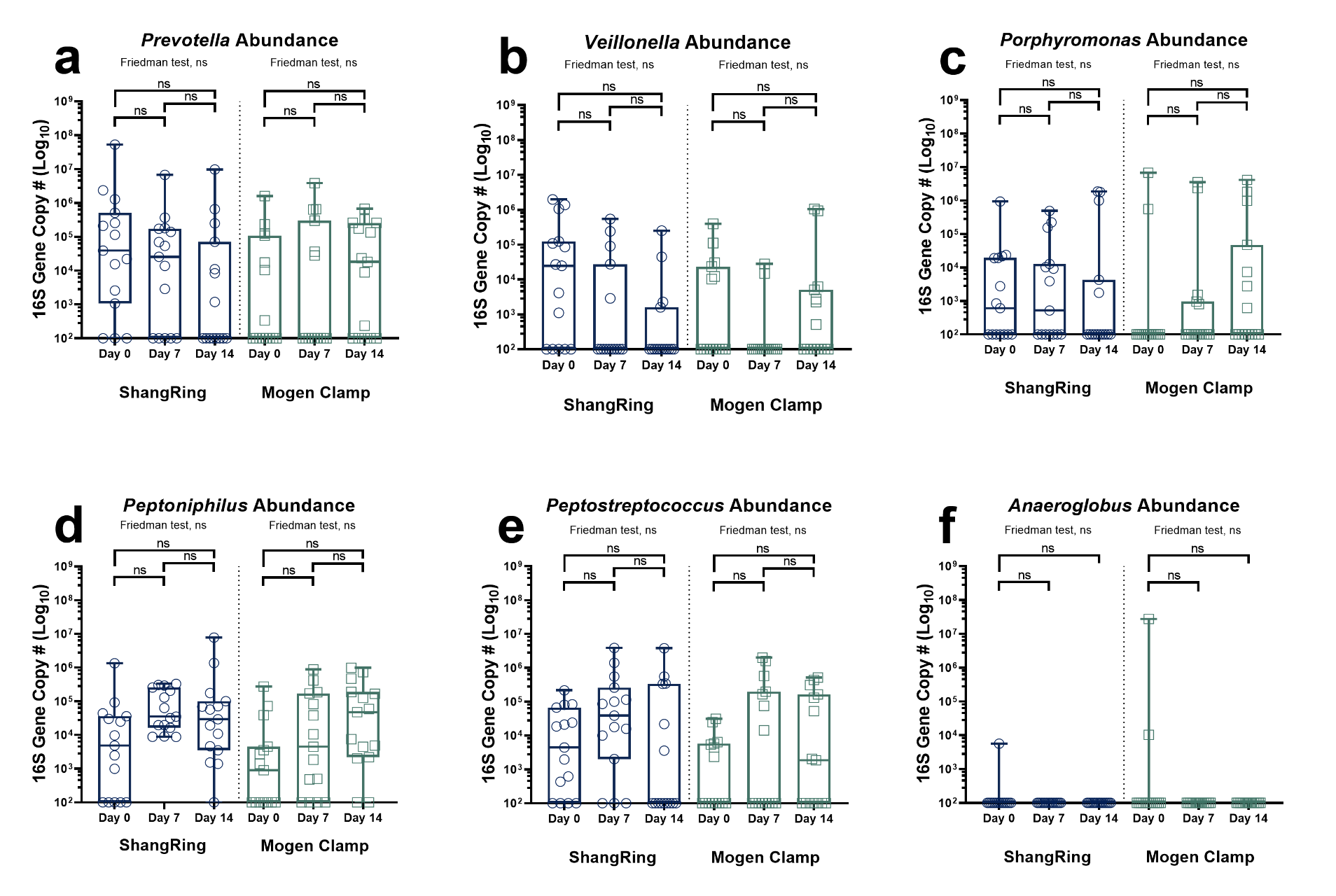


**Figure S1.** Taxon-specific absolute abundance changes in genital-associated anaerobes on the coronal sulcus by treatment arm (ShangRing = blue/left; Mogen Clamp = green/right) over time. Box plot denotes median and interquartile range with whisker signifying maximum and minimum values. Absolute abundance changes over time within-subjects were assessed using omnibus Friedman tests followed by pairwise Wilcoxon matched pair signed rank tests. P-values were corrected for the false discovery rate using the Benjamini Hochberg procedure. No significant bacterial load changes were observed in *Prevotella* (a), *Veillonella* (b), *Porphyromonas* (c), *Peptoniphilus* (d), *Peptostreptococcus* (e), or *Anaeroglobus* (f).

**Figure S2.** Enteric uropathogen taxon-specific density changes in the coronal sulcus by treatment arm over time.


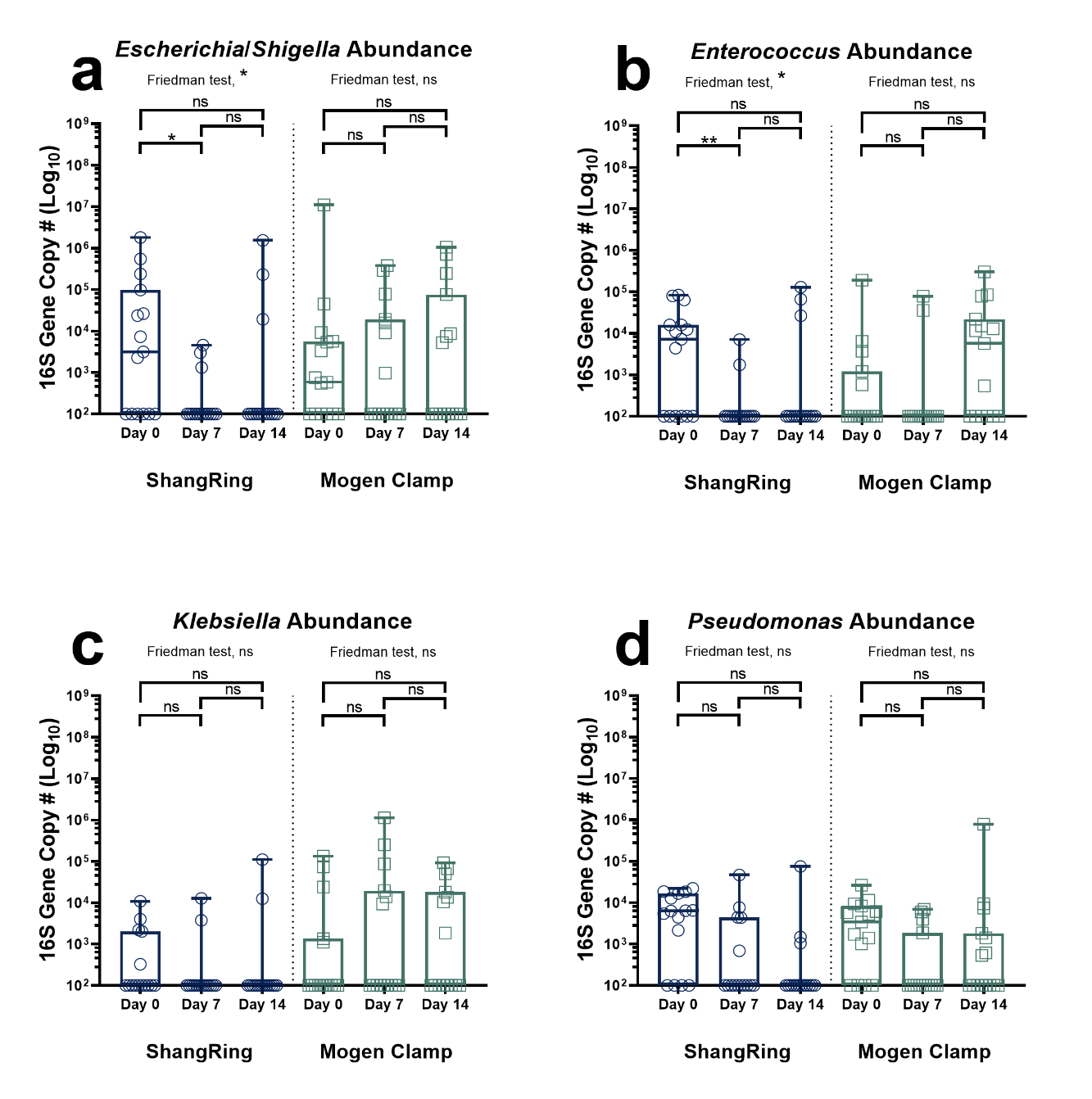


**Figure S2.** Taxon-specific absolute abundance changes in enteric uropathogens on the coronal sulcus by treatment arm (ShangRing = blue/left; Mogen Clamp = green/right) over time. Box plot denotes median and interquartile range with whisker signifying maximum and minimum values. Absolute abundance changes over time within-subjects were assessed using omnibus Friedman tests followed by pairwise Wilcoxon matched pair signed rank tests. P-values were corrected for the false discovery rate using the Benjamini Hochberg procedure. No significant bacterial load changes were observed in *Klebsiella* (c) or *Pseudomonas* (d). *Eschericihia/Shigella* (a) and *Enterococcus* (b) bacterial load decreased significantly in the ShangRing treatment arm. **p* <0.05. ***p*<0.01.

**Figure S3.** Taxon-specific density changes in the coronal sulcus by treatment arm over time.


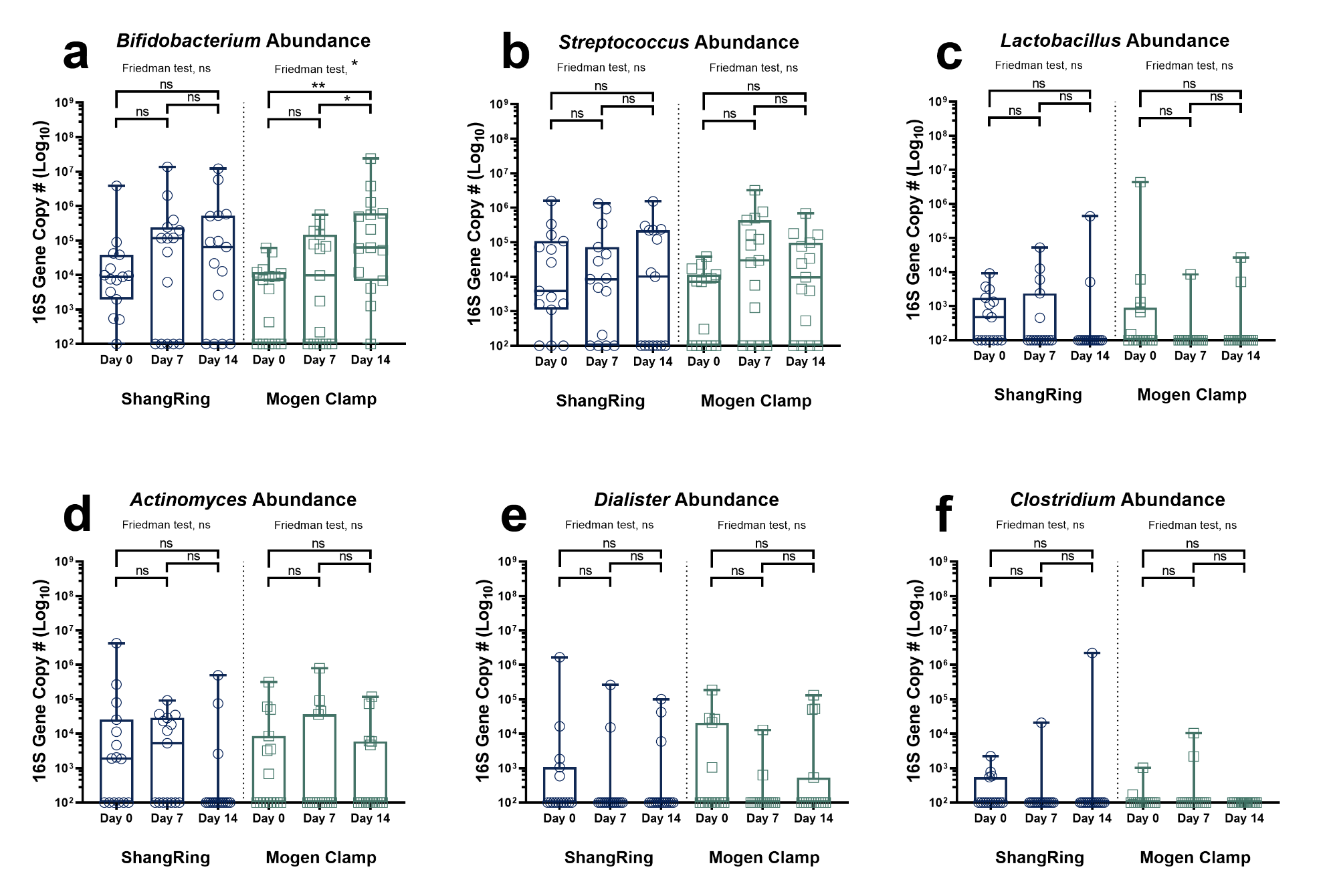


**Figure S3.** Taxon-specific absolute abundance changes in prevalent bacteria on the coronal sulcus by treatment arm (ShangRing = blue/left; Mogen Clamp = green/right) over time. Box plot denotes median and interquartile range with whisker signifying maximum and minimum values. Absolute abundance changes over time within-subjects were assessed using omnibus Friedman tests followed by pairwise Wilcoxon matched pair signed rank tests. P-values were corrected for the false discovery rate using the Benjamini Hochberg procedure. No significant bacterial load changes were observed in *Streptococcus* (b), *Lactobacillus* (c), *Actinomyces* (d), *Dialister* (e), or *Clostridium* (f). *Bifidobacterium* (a) bacterial load increased significantly in the Mogen clamp treatment arm. **p* <0.05. ***p*<0.01.

**Table S1**. Prevalence and Proportional Abundance of the 20 Most Prevalent Penile Bacteria in Uncircumcised Infants by Treatment Arm at Enrolment

|  |  |  | Prevalence in group, n (%) | | | | Average proportional abundance in group, % (SD) | | | |
| --- | --- | --- | --- | --- | --- | --- | --- | --- | --- | --- |
| Genus | Oxygen tolerance and pathogenicity ^a^ | Body sites ^b^ | ShangRing (n=15) | Mogen Clamp (n=15) | P-value ^c^ | ShangRing  (n = 15) | | Mogen Clamp  (n = 15) | P-value ^d^ |  |
| *Corynebacterium* | FAN | Skin, genital | 15 (100) | 15 (100) | - | 15.7 (16.1) | | 14.9 (15.0) | 0.95 |  |
| *Staphylococcus* | FAN | Skin, genital | 13 (86.7) | 12 (80.0) | 0.89 | 7.3 (7.1) | | 13.8 (19.0) | 0.95 |  |
| *Prevotella* | AN | Genital, gut | 13 (86.7) | 8 (53.3) | 0.38 | 16.4 (19.2) | | 5.3 (9.8) | 0.60 |  |
| *Veillonella* | AN | Genital, gut | 10 (66.7) | 7 (46.7) | 0.73 | 11.4 (17.3) | | 4.1 (9.6) | 0.88 |  |
| *Finegoldia* | AN | Skin, genital, gut | 11 (73.3) | 11 (73.3) | 1.0 | 1.3 (1.3) | | 3 (6.4) | 0.95 |  |
| *Porphyromonas* | AN | Genital | 8 (53.3) | 2 (13.3) | 0.38 | 0.9 (1.3) | | 2.8 (8.4) | 0.80 |  |
| *Peptoniphilus* | AN | Genital, gut | 10 (66.7) | 8 (53.3) | 0.80 | 1.6 (2.2) | | 0.9 (1.4) | 0.90 |  |
| *Anaerococcus* | AN | Skin, genital, gut | 12 (80.0) | 12 (80.0) | 1.0 | 0.8 (1.0) | | 2.0 (2.9) | 0.92 |  |
| *Peptostreptococcus* | AN | Genital | 11 (73.3) | 7 (46.7) | 0.67 | 1.6 (2.8) | | 0.8 (1.1) | 0.92 |  |
| *Dialister* | AN | Genital | 5 (33.3) | 5 (33.3) | 1.0 | 0.4 (0.7) | | 0.3 (0.7) | 0.95 |  |
| *Anaeroglobus* | AN | Genital | 1 (6.7) | 2 (13.3) | 0.86 | 0.04 (0.2) | | 3.2 (11.7) | 0.92 |  |
| *Escherichia* | FAN/UP | Gut | 9 (60.0) | 10 (66.7) | 0.89 | 6.4 (15.6) | | 2.2 (4.9) | 0.95 |  |
| *Enterococcus* | FAN/UP | Gut | 9 (60.0) | 6 (40.0) | 0.73 | 1.6 (3.8) | | 0.2 (0.3) | 0.88 |  |
| *Klebsiella* | FAN/UP | Gut | 5 (33.3) | 5 (33.3) | 1.0 | 0.3 (0.6) | | 6.2 (15.0) | 0.95 |  |
| *Pseudomonas* | FAN/AN/MAE/UP | Skin, gut | 11 (73.3) | 12 (80.0) | 0.89 | 2.2 (3.8) | | 1.5 (2.4) | 0.95 |  |
| *Bifidobacterium* | AN | Genital, gut | 14 (93.3) | 10 (66.7) | 0.44 | 2.5 (4.0) | | 4.1 (9.1) | 0.95 |  |
| *Streptococcus* | FAN | Gut | 12 (80.0) | 9 (60.0) | 0.73 | 7.5 (11.7) | | 2.4 (3.5) | 0.88 |  |
| *Lactobacillus* | FAN/AN/MAE | Skin, genital, gut | 8 (53.3) | 6 (40.0) | 0.79 | 0.4 (0.6) | | 5.1 (18.8) | 0.92 |  |
| *Actinomyces* | FAN | Gut | 9 (60.0) | 7 (46.7) | 0.79 | 1.5 (2.8) | | 0.9 (1.6) | 0.92 |  |
| *Clostridium* | AN | Gut | 4 (26.7) | 2 (13.3) | 0.79 | 0.04 (0.1) | | 0.01 (0.04) | 0.92 |  |

**Table S2.** Prevalence and Change in Prevalence of the 20 Most Prevalent Penile Bacteria by Treatment Arm 7- and 14-days Post-Enrolment

|  | Prevalence in group, n (%) ^a^ | | | | | | Change in Prevalence, % ^b^ | | | | | | | |
| --- | --- | --- | --- | --- | --- | --- | --- | --- | --- | --- | --- | --- | --- | --- |
|  | 7 Days | | | 14 Days | | | Day 0 to Day 7 | | | Day 7 to Day 14 | | | Day 0 to Day 14 | |
| Genus | ShangRing (n=15) | Mogen Clamp (n=15) | ShangRing  (n=15) | | Mogen Clamp (n=14) | ShangRing  (n=15) | | Mogen Clamp (n=15) | ShangRing  (n=15) | | Mogen Clamp  (n=14) | ShangRing  (n=15) | | Mogen Clamp  (n=14) |
| *Corynebacterium* | 15 (100) | 15 (100) | 15 (100) | | 14 (100) | 0.0 | | 0.0 | 0.0 | | 0.0 | 0.0 | | 0.0 |
| *Staphylococcus* | 15 (100) | 15 (100) | 15 (100) | | 14 (100) | 13.3 | | 20.0 | 0.0 | | 0.0 | 13.3 | | 20.0 |
| *Prevotella* | 10 (66.7) | 6 (40.0) | 7 (46.7) | | 10 (71.4) | -20.0 | | -13.3 | -20.0 | | 31.4 | -40.0 | | 18.1 |
| *Veillonella* | 5 (33.3) | 2 (13.3) | 4 (26.7) | | 7 (50.0) | -33.4 | | -33.4 | -6.6 | | 36.7 | -40.0 | | 3.3 |
| *Finegoldia* | 15 (100) | 13 (86.7) | 15 (100) | | 14 (100) | 26.7 | | 13.4 | 0.0 | | 13.3 | 26.7 | | 26.7 |
| *Porphyromonas* | 8 (53.3) | 5 (33.3) | 5 (33.3) | | 7 (50.0) | 0.0 | | 20.0 | -20.0 | | 16.7 | -20.0 | | 36.7 |
| *Peptoniphilus* | 15 (100) | 11 (73.3) | 14 (93.3) | | 12 (85.7) | 33.3 | | 20.0 | -6.7 | | 12.4 | 26.6 | | 32.4 |
| *Anaerococcus* | 15 (100) | 12 (80.0) | 14 (93.3) | | 13 (92.9) | 20.0 | | 0.0 | -6.7 | | 12.9 | 13.3 | | 12.9 |
| *Peptostreptococcus* | 12 (80) | 7 (46.7) | 6 (40.0) | | 8 (57.1) | 6.7 | | 0.0 | -40.0 | | 10.4 | -33.3 | | 10.4 |
| *Dialister* | 2 (13.3) | 2 (13.3) | 4 (26.7) | | 3 (21.4) | -20.0 | | -20.0 | 13.4 | | 8.1 | -6.7 | | -11.9 |
| *Anaeroglobus* | 0 (0.0) | 0 (0.0) | 0 (0.0) | | 0 (0.0) | -6.7 | | -13.3 | 0.0 | | 0.0 | -6.7 | | -13.3 |
| *Escherichia* | 3 (20.0) | 7 (46.7) | 3 (20.0) | | 7 (50.0) | -40.0 | | -20.0 | 0.0 | | 3.3 | -40.0 | | -16.7 |
| *Enterococcus* | 2 (13.3) | 2 (13.3) | 3 (20.0) | | 8 (57.1) | -46.7 | | -26.7 | 6.7 | | 43.8 | -40.0 | | 17.1 |
| *Klebsiella* | 2 (13.3) | 6 (40.0) | 2 (13.3) | | 7 (50.0) | -20.0 | | 6.7 | 0.0 | | 10.0 | -20.0 | | 16.7 |
| *Pseudomonas* | 5 (33.3) | 4 (26.7) | 3 (20.0) | | 7 (50.0) | -40.0 | | -53.3 | -13.3 | | 23.3 | -53.3 | | -30.0 |
| *Bifidobacterium* | 10 (66.7) | 10 (66.7) | 11 (73.3) | | 13 (92.9) | -26.6 | | 0.0 | 6.6 | | 26.2 | -20.0 | | 26.2 |
| *Streptococcus* | 11 (73.3) | 11 (73.3) | 8 (53.3) | | 10 (71.4) | -6.7 | | 13.3 | -20.0 | | -1.9 | -26.7 | | 11.4 |
| *Lactobacillus* | 5 (33.3) | 1 (6.7) | 2 (13.3) | | 2 (14.3) | -20.0 | | -33.3 | -20.0 | | 7.6 | -40.0 | | -25.7 |
| *Actinomyces* | 8 (53.3) | 4 (26.7) | 3 (20.0) | | 5 (35.7) | -6.7 | | -20.0 | -33.3 | | 9.0 | -40.0 | | -11.0 |
| *Clostridium* | 1 (6.7) | 2 (13.3) | 1 (6.7) | | 0 (0.0) | -20.0 | | 0.0 | 0.0 | | -13.3 | -20.0 | | -13.3 |

^a^ Significance testing for prevalence differences across treatment arms was done using Chi-square test. Adjusted for multiple comparisons using Benjamini-Hochberg correction.

^b^ Change in Prevalence is a percent change calculated by the change in prevalence observed in a single treatment group over time. Significance testing for within arm changes was done using McNemar’s test. Adjusted for multiple comparisons using Benjamini-Hochberg correction.

**Table S3.** Average Proportional Abundance and Change in Proportional Abundance of the 20 Most Prevalent Penile Bacteria by Treatment Arm 7- and 14-days Post-Enrolment

|  | Average proportional abundance in group, % (SD) ^a^ | | | | Change in Proportional abundance in group, % ^b^ | | | | | |
| --- | --- | --- | --- | --- | --- | --- | --- | --- | --- | --- |
|  | Day 7 | | Day 14 | | Day 0 to Day 7 | | Day 7 to Day 14 | | Day 0 to Day 14 | |
| Genus | ShangRing  (n=15) | Mogen Clamp  (n=15) | ShangRing  (n=15) | Mogen Clamp  (n=14) | ShangRing  (n=15) | Mogen Clamp  (n=15) | ShangRing  (n=15) | Mogen Clamp  (n=14) | ShangRing  (n=15) | Mogen Clamp  (n=14) |
| *Corynebacterium* | 19.7 (14.5) | 13.6 (13.1) | 40.7 (20.5) | 20.6 (14.0)* | 4.0 | -1.3 | 20.9 | 7.0 | 24.9* | 5.7 |
| *Staphylococcus* | 42.7 (29.3) | 56.3 (28.1) | 28.4 (17.4) | 31.0 (23.7) | 35.5** | 42.5* | -14.3 | -25.3 | 21.2** | 17.2 |
| *Prevotella* | 4.3 (10.9) | 1.6 (3.4) | 1.5 (3.5) | 2.1 (2.8) | -12.1 | -3.7 | -2.9 | 0.5 | -15.0* | -3.3 |
| *Veillonella* | 1.3 (4.3) | 0.1 (0.2) | 0.1 (0.1) | 1.9 (4.8) | -10.1 | -4.1 | -1.2 | 1.8 | -11.3* | -2.3 |
| *Finegoldia* | 2.5 (2.9) | 2.4 (3.8) | 3.0 (4.5) | 6.4 (8.3) | 1.3 | -0.6 | 0.5 | 4.0 | 1.7 | 3.4 |
| *Porphyromonas* | 0.9 (1.5) | 1.3 (3.4) | 2.4 (5.2) | 4.7 (10.8) | -0.1 | -1.6 | 1.6 | 3.4 | 1.5 | 1.8 |
| *Peptoniphilus* | 1.8 (2.2) | 1.1 (2.2) | 1.6 (3.4) | 2.7 (3.2) | 0.3 | 0.2 | -0.3 | 1.5 | 0.0 | 1.8 |
| *Anaerococcus* | 4.8 (5.6) | 2.1 (3.3) | 2.1 (2.8) | 5.0 (5.5) | 4.1 | 0.1 | -2.7 | 2.9 | 1.4 | 3.0 |
| *Peptostreptococcus* | 3.3 (6.5) | 1.9 (3.9) | 0.9 (1.7) | 1.9 (2.5) | 1.7 | 1.1 | -2.4 | -0.1 | -0.7 | 1.0 |
| *Dialister* | 0.02 (0.1) | 0.1 (0.2) | 0.1 (0.3) | 0.1 (0.3) | -0.4 | -0.3 | 0.1 | 0.0 | -0.3 | -0.2 |
| *Anaeroglobus* | 0.0 (0.0) | 0.0 (0.0) | 0.0 (0.0) | 0.0 (0.0) | 0.0 | -3.2 | 0.0 | 0.0 | 0.0 | -3.2 |
| *Escherichia* | 0.1 (0.1) | 1.3 (3.1) | 0.9 (3.5) | 1.2 (2.5) | -6.3 | -0.9 | 0.9 | -0.2 | -5.4 | -1.1 |
| *Enterococcus* | 0.01 (0.04) | 0.1 (0.4) | 0.1 (0.3) | 0.4 (0.8) | -1.6 | -0.1 | 0.1 | 0.3 | -1.5* | 0.2 |
| *Klebsiella* | 0.03 (0.1) | 4.2 (11.5) | 0.1 (0.3) | 0.4 (1.2) | -0.2 | -1.9 | 0.1 | -3.8 | -0.2 | -5.7 |
| *Pseudomonas* | 0.1 (0.3) | 0.2 (0.4) | 0.1 (0.3) | 1.6 (5.6) | -2.1 | -1.3 | 0.0 | 1.4 | -2.1* | 0.1 |
| *Bifidobacterium* | 7.5 (12.8) | 0.8 (1.1) | 4.3 (9.0) | 12.4 (22.5) | 5.0 | -3.3 | -3.1 | 11.6** | 1.9 | 8.3 |
| *Streptococcus* | 2.2 (5.2) | 2.5 (5.2) | 2.1 (4.8) | 1.7 (3.1) | -5.3 | 0.1 | -0.1 | -0.8 | -5.4 | -0.8 |
| *Lactobacillus* | 0.1 (0.1) | 0.1 (0.1) | 0.1 (0.2) | 0.04 (0.1) | -0.3 | -5.0 | 0.0 | 0.0 | -0.3 | -5.0 |
| *Actinomyces* | 0.2 (0.3) | 0.4 (0.9) | 0.1 (0.2) | 0.2 (0.4) | -1.3 | -0.5 | -0.1 | -0.2 | -1.4 | -0.7 |
| *Clostridium* | 0.01 (0.1) | 0.01 (0.03) | 0.2 (0.8) | 0.0 (0.0) | 0.0 | 0.0 | 0.2 | 0.0 | 0.2 | 0.0 |

^a^ Significance testing for proportional abundance differences across treatment arms was done using Mann-Whitney U test. Adjusted for multiple comparisons using Benjamini-Hochberg correction.

^b^  Change in proportional abundance is a percentile calculated by the change in proportional abundance observed in a single treatment group over time. Significance testing for within arm changes was done using Wilcoxon matched pairs signed-rank test. Adjusted for multiple comparisons using Benjamini-Hochberg correction.

**p* <0.05. ***p*<0.01.

**Table S4.** Median Absolute Abundance of the 20 Most Prevalent Penile Bacteria by Treatment Arm 7- and 14-days Post-Enrolment

|  | Median absolute abundance [IQR] in group ^a^ | | | | | |
| --- | --- | --- | --- | --- | --- | --- |
|  | Baseline | | Day 7 | | Day 14 | |
| Genus | ShangRing  (n=15) | Mogen Clamp  (n=15) | ShangRing  (n=15) | Mogen Clamp  (n=15) | ShangRing  (n=15) | Mogen Clamp  (n=14) |
| Total | 6.1 x 10^5^ [2.8 x 10^5^ - 2.7 x 10^6^] | 2.9 x 10^5^ [2.5 x 10^5^ - 1.1 x 10^6^] | 1.1 x 10^7^ [2.1 x 10^6^ - 2.3 x 10^7^] | 1.2 x 10^7^ [2.3 x 10^6^ - 2.6 x 10^7^] | 1.1 x 10^7^ [5.9 x 10^6^ - 1.8 x 10^7^] | 3.7 x 10^6^ [1.5 x 10^6^ - 1.1 x 10^7^] |
| *Corynebacterium* | 6.1 x 10^4^ [2.8 x 10^4^ - 1.9 x 10^5^] | 2.5 x 10^4^ [7.2 x 10^3^ - 1.1 x 10^5^] | 1.7 x 10^6^ [9.3 x 10^5^- 2.6 x 10^6^] | 6.4 x 10^5^ [2.5 x 10^5^ - 1.9 x 10^6^] | 5.1 x 10^6^ [1.8 x 10^6^ - 7.4 x 10^6^] | 7.7 x 10^5^ [2.3 x 10^5^ - 1.6 x 10^6^] |
| *Staphylococcus* | 6.8 x 10^4^ [1.1 x 10^4^ - 1.1 x 10^5^] | 2.5 x 10^4^ [8.5 x 10^2^ - 5.3 x 10^4^] | 3.5 x 10^6^ [3.1 x 10^5^- 1.2 x 10^7^] | 5.3 x 10^6^ [1.0 x 10^6^ - 1.2 x 10^7^] | 2.6 x 10^6^ [1.1 x 10^6^ - 4.3 x 10^6^] | 1.2 x 10^6^ [5.0 x 10^5^ - 1.9 x 10^6^] |
| *Prevotella* | 3.9 x 10^4^ [1.8 x 10^3^ - 3.8 x 10^5^] | 0.0 x 10^0^ [0.0 x 10^0^ - 6.3 x 10^4^]* | 2.6 x 10^4^ [0.0 x 10^0^- 1.6 x 10^5^] | 0.0 x 10^0^ [0.0 x 10^0^ - 1.7 x 10^5^] | 0.0 x 10^0^ [0.0 x 10^0^ - 4.1 x 10^4^] | 2.1 x 10^4^ [5.9 x 10^1^ - 2.4 x 10^5^] |
| *Veillonella* | 2.5 x 10^4^ [0.0 x 10^0^ - 1.2 x 10^5^] | 0.0 x 10^0^ [0.0 x 10^0^ - 1.8 x 10^4^] | 0.0 x 10^0^ [0.0 x 10^0^- 1.5 x 10^4^] | 0.0 x 10^0^ [0.0 x 10^0^ - 0.0 x 10^0^] | 0.0 x 10^0^ [0.0 x 10^0^ - 8.0 x 10^2^] | 2.6 x 10^2^ [0.0 x 10^0^ - 4.5 x 10^3^] |
| *Finegoldia* | 7.5 x 10^3^ [4.1 x 10^1^ - 2.2 x 10^4^] | 1.7 x 10^3^ [8.7 x 10^1^ - 1.2 x 10^4^] | 1.4 x 10^5^ [2.5 x 10^4^- 2.9 x 10^5^] | 5.0 x 10^4^ [1.6 x 10^4^ - 1.7 x 10^5^] | 1.4 x 10^5^ [2.4 x 10^4^ - 4.1 x 10^5^] | 1.9 x 10^5^ [4.5 x 10^4^ - 3.8 x 10^5^] |
| *Porphyromonas* | 6.1 x 10^2^ [0.0 x 10^0^ - 1.9 x 10^4^] | 0.0 x 10^0^ [0.0 x 10^0^ - 0.0 x 10^0^] | 5.2 x 10^2^ [0.0 x 10^0^- 1.1 x 10^4^] | 0.0 x 10^0^ [0.0 x 10^0^ - 8.8 x 10^2^] | 0.0 x 10^0^ [0.0 x 10^0^ - 3.0 x 10^3^] | 3.1 x 10^2^ [0.0 x 10^0^ - 3.7 x 10^4^] |
| *Peptoniphilus* | 4.9 x 10^3^ [0.0 x 10^0^ - 3.3 x 10^4^] | 9.0 x 10^2^ [0.0 x 10^0^ - 4.0 x 10^3^] | 3.5 x 10^4^ [1.8 x 10^4^- 2.3 x 10^5^] | 4.5 x 10^3^ [2.4 x 10^2^ - 1.3 x 10^5^] | 2.9 x 10^4^ [4.1 x 10^3^ - 8.6 x 10^4^] | 5.3 x 10^4^ [2.9 x 10^3^ - 1.8 x 10^5^] |
| *Anaerococcus* | 1.8 x 10^3^ [3.4 x 10^2^ - 1.6 x 10^4^] | 3.9 x 10^3^ [4.5 x 10^2^ - 6.6 x 10^3^] | 1.4 x 10^5^ [5.4 x 10^4^- 3.7 x 10^5^] | 4.3 x 10^4^ [1.5 x 10^3^ - 1.1 x 10^5^] | 4.0 x 10^4^ [1.0 x 10^4^ - 3.6 x 10^5^] | 1.1 x 10^5^ [2.7 x 10^4^ - 3.5 x 10^5^] |
| *Peptostreptococcus* | 4.4 x 10^3^ [2.2 x 10^2^ - 4.6 x 10^4^] | 0.0 x 10^0^ [0.0 x 10^0^ - 5.6 x 10^3^] | 3.9 x 10^4^ [6.0 x 10^3^- 1.9 x 10^5^] | 0.0 x 10^0^ [0.0 x 10^0^ - 1.8 x 10^5^] | 0.0 x 10^0^ [0.0 x 10^0^ - 1.8 x 10^5^] | 1.9 x 10^3^ [0.0 x 10^0^ - 1.6 x 10^5^] |
| *Dialister* | 0.0 x 10^0^ [0.0 x 10^0^ - 1.1 x 10^4^] | 0.0 x 10^0^ [0.0 x 10^0^ - 8.3 x 10^2^] | 0.0 x 10^0^ [0.0 x 10^0^- 0.0 x 10^0^] | 0.0 x 10^0^ [0.0 x 10^0^ - 0.0 x 10^0^] | 0.0 x 10^0^ [0.0 x 10^0^ - 2.6 x 10^2^] | 0.0 x 10^0^ [0.0 x 10^0^ - 0.0 x 10^0^] |
| *Anaeroglobus* | 0.0 x 10^0^ [0.0 x 10^0^ - 0.0 x 10^0^] | 0.0 x 10^0^ [0.0 x 10^0^ - 0.0 x 10^0^] | 0.0 x 10^0^ [0.0 x 10^0^- 0.0 x 10^0^] | 0.0 x 10^0^ [0.0 x 10^0^ - 0.0 x 10^0^] | 0.0 x 10^0^ [0.0 x 10^0^ - 0.0 x 10^0^] | 0.0 x 10^0^ [0.0 x 10^0^ - 0.0 x 10^0^] |
| *Escherichia* | 3.2 x 10^3^ [0.0 x 10^0^ - 6.2 x 10^4^] | 5.8 x 10^2^ [0.0 x 10^0^ - 5.6 x 10^3^] | 0.0 x 10^0^ [0.0 x 10^0^- 0.0 x 10^0^] | 0.0 x 10^0^ [0.0 x 10^0^ - 1.7 x 10^4^] | 0.0 x 10^0^ [0.0 x 10^0^ - 0.0 x 10^0^] | 2.6 x 10^3^ [0.0 x 10^0^ - 5.9 x 10^4^] |
| *Enterococcus* | 7.2 x 10^3^ [0.0 x 10^0^ - 1.6 x 10^4^] | 0.0 x 10^0^ [0.0 x 10^0^ - 8.9 x 10^2^] | 0.0 x 10^0^ [0.0 x 10^0^- 0.0 x 10^0^] | 0.0 x 10^0^ [0.0 x 10^0^ - 0.0 x 10^0^] | 0.0 x 10^0^ [0.0 x 10^0^ - 0.0 x 10^0^] | 3.1 x 10^3^ [0.0 x 10^0^ - 1.5 x 10^4^] |
| *Klebsiella* | 0.0 x 10^0^ [0.0 x 10^0^ - 1.2 x 10^3^] | 0.0 x 10^0^ [0.0 x 10^0^ - 1.2 x 10^3^] | 0.0 x 10^0^ [0.0 x 10^0^- 0.0 x 10^0^] | 0.0 x 10^0^ [0.0 x 10^0^ - 1.7 x 10^4^] | 0.0 x 10^0^ [0.0 x 10^0^ - 0.0 x 10^0^] | 9.3 x 10^2^ [0.0 x 10^0^ - 1.7 x 10^4^] |
| *Pseudomonas* | 6.3 x 10^3^ [1.1 x 10^3^ - 1.5 x 10^4^] | 3.4 x 10^3^ [5.0 x 10^2^ - 7.2 x 10^3^] | 0.0 x 10^0^ [0.0 x 10^0^- 2.5 x 10^3^] | 0.0 x 10^0^ [0.0 x 10^0^ - 9.3 x 10^2^] | 0.0 x 10^0^ [0.0 x 10^0^ - 0.0 x 10^0^] | 2.6 x 10^2^ [0.0 x 10^0^ - 1.7 x 10^3^] |
| *Bifidobacterium* | 8.9 x 10^3^ [2.6 x 10^3^ - 2.7 x 10^4^] | 7.3 x 10^3^ [0.0 x 10^0^ - 1.2 x 10^4^] | 1.2 x 10^5^ [0.0 x 10^0^- 2.2 x 10^5^] | 9.7 x 10^3^ [0.0 x 10^0^ - 1.1 x 10^5^] | 6.5 x 10^4^ [1.3 x 10^3^ - 5.2 x 10^5^] | 6.1 x 10^4^ [8.1 x 10^3^ - 5.9 x 10^5^] |
| *Streptococcus* | 3.9 x 10^3^ [1.4 x 10^3^ - 9.0 x 10^4^] | 7.2 x 10^3^ [0.0 x 10^0^ - 1.0 x 10^4^] | 8.6 x 10^3^ [1.0 x 10^2^- 5.9 x 10^4^] | 3.0 x 10^4^ [6.3 x 10^2^ - 3.0 x 10^5^] | 1.0 x 10^4^ [0.0 x 10^0^ - 2.2 x 10^5^] | 7.1 x 10^3^ [1.3 x 10^2^ - 6.5 x 10^4^] |
| *Lactobacillus* | 4.7 x 10^2^ [0.0 x 10^0^ - 1.6 x 10^3^] | 0.0 x 10^0^ [0.0 x 10^0^ - 7.9 x 10^2^] | 0.0 x 10^0^ [0.0 x 10^0^- 1.4 x 10^3^] | 0.0 x 10^0^ [0.0 x 10^0^ - 0.0 x 10^0^] | 0.0 x 10^0^ [0.0 x 10^0^ - 0.0 x 10^0^] | 0.0 x 10^0^ [0.0 x 10^0^ - 0.0 x 10^0^] |
| *Actinomyces* | 1.9 x 10^3^ [0.0 x 10^0^ - 1.9 x 10^4^] | 0.0 x 10^0^ [0.0 x 10^0^ - 6.1 x 10^3^] | 5.3 x 10^3^ [0.0 x 10^0^- 2.6 x 10^4^] | 0.0 x 10^0^ [0.0 x 10^0^ - 1.8 x 10^4^] | 0.0 x 10^0^ [0.0 x 10^0^ - 0.0 x 10^0^] | 0.0 x 10^0^ [0.0 x 10^0^ - 5.7 x 10^3^] |
| *Clostridium* | 0.0 x 10^0^ [0.0 x 10^0^ - 2.8 x 10^2^] | 0.0 x 10^0^ [0.0 x 10^0^ - 0.0 x 10^0^] | 0.0 x 10^0^ [0.0 x 10^0^- 0.0 x 10^0^] | 0.0 x 10^0^ [0.0 x 10^0^ - 0.0 x 10^0^] | 0.0 x 10^0^ [0.0 x 10^0^ - 0.0 x 10^0^] | 0.0 x 10^0^ [0.0 x 10^0^ - 0.0 x 10^0^] |

^a^ Significance testing for absolute abundance differences across treatment arms was done using Mann-Whitney U test. Adjusted for multiple comparisons using Benjamini-Hochberg correction.

**p* <0.05.

**Table S5.** Median Proportional Abundance of the 20 Most Prevalent Penile Bacteria by Treatment Arm 7- and 14-days Post-Enrolment

|  | Median proportional abundance in group, % [IQR] ^a^ | | | | | |
| --- | --- | --- | --- | --- | --- | --- |
|  | Day 0 | | Day 7 | | Day 14 | |
| Genus | ShangRing  (n=15) | Mogen Clamp  (n=15) | ShangRing  (n=15) | Mogen Clamp  (n=15) | ShangRing  (n=15) | Mogen Clamp  (n=14) |
| *Corynebacterium* | 11.8 [2.7 - 25.0] | 15.9 [2.4 - 21.0] | 12.1 [10.6 - 22.8] | 7.2 [3.5 - 22.7] | 42.2 [28.0 - 53.8] | 20.9 [9.4 - 28.4]* |
| *Staphylococcus* | 7.1 [1.5 - 10.0] | 4.8 [1.3 - 18.9] | 38.5 [19.6 - 65.2] | 58.9 [42.1 - 75.7] | 20.6 [18.1 - 36.2] | 24.6 [16.3 - 38.6] |
| *Prevotella* | 13.8 [1.2 - 26.2] | 0.0 [0.0 - 5.8] | 0.3 [0.0 - 1.6] | 0.0 [0.0 - 1.7] | 0.0 [0.0 - 0.3] | 0.5 [0.0 - 2.8] |
| *Veillonella* | 3.4 [0.0 - 14.4] | 0.0 [0.0 - 2.0] | 0.0 [0.0 - 0.2] | 0.0 [0.0 - 0.0] | 0.0 [0.0 - 0.1] | 0.0 [0.0 - 0.2] |
| *Finegoldia* | 1.0 [0.0 - 2.5] | 0.4 [0.0 - 2.6] | 1.6 [0.4 - 3.6] | 0.9 [0.3 - 2.4] | 1.5 [0.3 - 2.5] | 3.9 [0.8 - 5.4] |
| *Porphyromonas* | 0.1 [0.0 - 1.6] | 0.0 [0.0 - 0.0] | 0.0 [0.0 - 1.0] | 0.0 [0.0 - 0.0] | 0.0 [0.0 - 0.2] | 0.0 [0.0 - 1.0] |
| *Peptoniphilus* | 0.8 [0.0 - 2.4] | 0.3 [0.0 - 0.9] | 0.9 [0.3 - 2.6] | 0.2 [0.0 - 1.2] | 0.3 [0.1 - 0.6] | 1.5 [0.1 - 4.0] |
| *Anaerococcus* | 0.4 [0.1 - 1.1] | 0.7 [0.2 - 2.2] | 1.6 [1.0 - 6.7] | 0.4 [0.1 - 2.1] | 1.0 [0.3 - 2.8] | 2.4 [0.9 - 7.8] |
| *Peptostreptococcus* | 0.3 [0.1 - 1.4] | 0.0 [0.0 - 1.6] | 0.4 [0.1 - 3.0] | 0.0 [0.0 - 1.3] | 0.0 [0.0 - 1.1] | 0.1 [0.0 - 4.0] |
| *Dialister* | 0.0 [0.0 - 0.5] | 0.0 [0.0 - 0.3] | 0.0 [0.0 - 0.0] | 0.0 [0.0 - 0.0] | 0.0 [0.0 - 0.0] | 0.0 [0.0 - 0.0] |
| *Anaeroglobus* | 0.0 [0.0 - 0.0] | 0.0 [0.0 - 0.0] | 0.0 [0.0 - 0.0] | 0.0 [0.0 - 0.0] | 0.0 [0.0 - 0.0] | 0.0 [0.0 - 0.0] |
| *Escherichia* | 0.3 [0.0 - 1.9] | 0.3 [0.0 - 1.0] | 0.0 [0.0 - 0.0] | 0.0 [0.0 - 0.8] | 0.0 [0.0 - 0.0] | 0.0 [0.0 - 0.7] |
| *Enterococcus* | 0.2 [0.0 - 1.1] | 0.0 [0.0 - 0.4] | 0.0 [0.0 - 0.0] | 0.0 [0.0 - 0.0] | 0.0 [0.0 - 0.0] | 0.1 [0.0 - 0.3] |
| *Klebsiella* | 0.0 [0.0 - 0.2] | 0.0 [0.0 - 0.8] | 0.0 [0.0 - 0.0] | 0.0 [0.0 - 2.1] | 0.0 [0.0 - 0.0] | 0.0 [0.0 - 0.3] |
| *Pseudomonas* | 1.3 [0.1 - 2.5] | 0.7 [0.2 - 1.6] | 0.0 [0.0 - 0.2] | 0.0 [0.0 - 0.1] | 0.0 [0.0 - 0.0] | 0.0 [0.0 - 0.1] |
| *Bifidobacterium* | 1.5 [0.3 - 2.0] | 0.5 [0.0 - 3.6] | 0.9 [0.0 - 7.7] | 0.3 [0.0 - 1.1] | 0.6 [0.1 - 3.1] | 2.8 [0.4 - 8.0] |
| *Streptococcus* | 2.0 [0.2 - 10.7] | 0.4 [0.0 - 3.2] | 0.2 [0.0 - 0.4] | 0.2 [0.1 - 2.5] | 0.1 [0.0 - 0.8] | 0.2 [0.0 - 1.9] |
| *Lactobacillus* | 0.1 [0.0 - 0.3] | 0.0 [0.0 - 0.1] | 0.0 [0.0 - 0.1] | 0.0 [0.0 - 0.0] | 0.0 [0.0 - 0.0] | 0.0 [0.0 - 0.0] |
| *Actinomyces* | 0.4 [0.0 - 1.3] | 0.0 [0.0 - 1.0] | 0.0 [0.0 - 0.2] | 0.0 [0.0 - 0.1] | 0.0 [0.0 - 0.0] | 0.0 [0.0 - 0.1] |
| *Clostridium* | 0.0 [0.0 - 0.0] | 0.0 [0.0 - 0.0] | 0.0 [0.0 - 0.0] | 0.0 [0.0 - 0.0] | 0.0 [0.0 - 0.0] | 0.0 [0.0 - 0.0] |

^a^ Significance testing for proportional abundance differences across treatment arms was done using Mann-Whitney U test. Adjusted for multiple comparisons using Benjamini-Hochberg correction.

**p* <0.05.

**Table S6.** Median Change in Proportional Abundance of the 20 Most Prevalent Penile Bacteria by Treatment Arm 7- and 14-days Post-Enrolment

|  | Change in Proportional abundance in group, % [IQR] ^a^ | | | | | |
| --- | --- | --- | --- | --- | --- | --- |
|  | Day 0 to Day 7 | | Day 7 to Day 14 | | Day 0 to Day 14 | |
| Genus | ShangRing  (n=15) | Mogen Clamp  (n=15) | ShangRing  (n=15) | Mogen Clamp  (n=14) | ShangRing  (n=15) | Mogen Clamp  (n=14) |
| *Corynebacterium* | 7.1 [-11.2 - 11.9] | 2.6 [-12.9 - 10.5] | 21.4 [6.4 - 40.1] | 10.6 [-9.1 - 21.6] | 21.7 [11.5 - 42.4]* | 7.0 [-2.9 - 16.7] |
| *Staphylococcus* | 31.4 [17.2 - 58.3]** | 48.6 [27.2 - 66.7]* | -16.3 [-35.8 - 4.5] | -27.7 [-47.9 - -4] | 17.6 [7.6 - 29.1]** | 18.9 [9.6 - 28.6] |
| *Prevotella* | -2.6 [-19.9 - 0.0] | 0.0 [-4.9 - 0.0] | -0.1 [-0.8 - 0.1] | 0.0 [-0.3 - 1.8] | -13.8 [-26.2 - 0.0]* | 0.0 [-4.6 - 2.7] |
| *Veillonella* | -2.6 [-14.4 - 0.0] | 0.0 [-2 - 0.0] | 0.0 [0.0 - 0.0] | 0.0 [0.0 - 0.0] | -3.4 [-14.4 - 0.0]* | -0.1 [-2.4 - 0.0] |
| *Finegoldia* | 0.7 [0.2 - 1.9] | 0.0 [-0.9 - 0.9] | -0.2 [-2.0 - 1.6] | 1.2 [0.1 - 4.6] | 0.9 [-1.2 - 1.8] | 1.7 [0.2 - 4.2] |
| *Porphyromonas* | 0.0 [-0.8 - 0.3] | 0.0 [0.0 - 0.0] | 0.0 [-0.1 - 0.1] | 0.0 [0.0 - 1.0] | 0.0 [-1.4 - 0.1] | 0.0 [0.0 - 1.0] |
| *Peptoniphilus* | 0.5 [-0.6 - 1.5] | 0.0 [-0.7 - 0.2] | -0.6 [-1.1 - 0.2] | 1.1 [-0.1 - 2.3] | 0.1 [-1.2 - 0.2] | 0.2 [0.0 - 1.5] |
| *Anaerococcus* | 1.3 [0.4 - 6.0] | -0.4 [-1.1 - 0.0] | -1.6 [-3.8 - 0.0] | 1.6 [0.5 - 3.6] | 0.4 [-0.3 - 2.0] | 1.7 [-0.1 - 5.6] |
| *Peptostreptococcus* | 0.3 [-0.8 - 2.9] | 0.0 [-0.5 - 0.5] | -0.3 [-1.0 - -0.1] | 0.0 [-0.5 - 3.0] | 0.0 [-0.9 - 0.6] | 0.0 [0.0 - 2.0] |
| *Dialister* | 0.0 [-0.5 - 0.0] | 0.0 [-0.3 - 0.0] | 0.0 [0.0 - 0.0] | 0.0 [0.0 - 0.0] | 0.0 [-0.4 - 0.0] | 0.0 [-0.3 - 0.0] |
| *Anaeroglobus* | 0.0 [0.0 - 0.0] | 0.0 [0.0 - 0.0] | 0.0 [0.0 - 0.0] | 0.0 [0.0 - 0.0] | 0.0 [0.0 - 0.0] | 0.0 [0.0 - 0.0] |
| *Escherichia* | -0.3 [-1.8 - 0.0] | 0.0 [-0.6 - 0.0] | 0.0 [0.0 - 0.0] | 0.0 [-1.2 - 0.1] | -0.2 [-1.8 - 0.0] | -0.2 [-0.7 - 0.1] |
| *Enterococcus* | -0.2 [-1.1 - 0.0] | 0.0 [-0.4 - 0.0] | 0.0 [0.0 - 0.0] | 0.0 [0.0 - 0.2] | -0.1 [-1.1 - 0.0]* | 0.0 [-0.2 - 0.3] |
| *Klebsiella* | 0.0 [-0.2 - 0.0] | 0.0 [-0.8 - 0.4] | 0.0 [0.0 - 0.0] | 0.0 [-0.2 - 0.0] | 0.0 [-0.1 - 0.0] | 0.0 [-1.2 - 0.0] |
| *Pseudomonas* | -0.6 [-2.5 - 0.0] | -0.7 [-1.6 - 0.0] | 0.0 [-0.2 - 0.0] | 0.0 [0.0 - 0.1] | -1.0 [-2.1 - -0.1]* | -0.7 [-1.5 - -0.1] |
| *Bifidobacterium* | 0.8 [-1.6 - 7.6] | -0.4 [-3.6 - 0.3] | 0.0 [-2.2 - 0.6] | 1.1 [0.2 - 7.8] | 0.3 [-1.9 - 2.7] | 0.4 [-1.9 - 5.1] |
| *Streptococcus* | -1.8 [-10.5 - 0.0] | -0.2 [-2.4 - 0.5] | 0.0 [-0.2 - 0.0] | 0.0 [-0.8 - 0.1] | -1.9 [-10.6 - 0.0] | -0.2 [-3.1 - 0.0] |
| *Lactobacillus* | 0.0 [-0.3 - 0.0] | 0.0 [-0.1 - 0.0] | 0.0 [0.0 - 0.0] | 0.0 [0.0 - 0.0] | -0.1 [-0.3 - 0.0] | 0.0 [-0.1 - 0.0] |
| *Actinomyces* | -0.4 [-1 - 0.0] | 0.0 [-1.0 - 0.0] | 0.0 [-0.2 - 0.0] | 0.0 [-0.1 - 0.0] | -0.4 [-1.3 - 0.0] | 0.0 [-1.0 - 0.0] |
| *Clostridium* | 0.0 [0.0 - 0.0] | 0.0 [0.0 - 0.0] | 0.0 [0.0 - 0.0] | 0.0 [0.0 - 0.0] | 0.0 [0.0 - 0.0] | 0.0 [0.0 - 0.0] |

^a^ Change in proportional abundance is a percentile calculated by the change in proportional abundance observed in a single treatment group over time. Significance testing for within arm changes was done using Wilcoxon matched pairs signed-rank test. Adjusted for multiple comparisons using Benjamini-Hochberg correction.

**p* <0.05. ***p*<0.01.
